# Investigating the relationship between attention-deficit hyperactivity disorder (ADHD) and C-reactive protein (CRP): observational, polygenic risk score, and Mendelian randomization analyses

**DOI:** 10.1017/S0033291725000480

**Published:** 2025-03-31

**Authors:** Yaxin Luo, Rachel Blakey, Apostolos Gkatzionis, Evie Stergiakouli, Christina Dardani

**Affiliations:** 1 Population Health Sciences, Bristol Medical School, University of Bristol, Bristol, UK; 2 Medical Research Council Integrative Epidemiology Unit, Bristol Medical School, University of Bristol, Bristol, UK; 3 Research Department, Lovisnberg Diaconal Hospital, Oslo, Norway; 4 PsychGen Centre for Genetic Epidemiology and Mental Health, Norwegian Institute of Public Health, Oslo, Norway

**Keywords:** ALSPAC, ADHD, systemic inflammation, cytokine, epidemiology, polygenic association, causal inference, triangulation

## Abstract

**Background:**

Emerging evidence suggests a co-occurrence of attention-deficit hyperactivity disorder (ADHD) and immune response-related conditions. However, it is unclear whether there is a causal relationship between ADHD and immune response.

**Methods:**

We investigated the associations between ADHD traits, common variant genetic liability to ADHD, and serum C-reactive protein (CRP) levels in childhood and adulthood, using data from the Avon Longitudinal Study of Parent and Children. Genetic correlation was estimated using linkage-disequilibrium score regression. Two-sample Mendelian randomization (MR) was conducted to test potential causal effects of ADHD genetic liability on serum CRP as an indicator of systemic inflammation, as well as the genetically proxied levels of specific plasma cytokines.

**Results:**

There was little evidence to suggest association between ADHD and CRP in childhood and adulthood. ADHD genetic liability was associated with a higher serum CRP at ages 9 (*β* = 0.02, 95% confidence interval [CI] = 0, 0.03), 15 (*β* = 0.04; 95% CI = 0.02, 0.06), and 24 years (*β* = 0.03; 95% CI = 0.01, 0.05). There was evidence of genetic correlations between ADHD and CRP ( 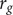 = 0.27; 95% CI = 0.19, 0.35). Evidence of a bidirectional effect of genetic liability to ADHD and CRP was found by two-sample MR (ADHD-CRP: *β*_IVW_= 0.04, 95% CI = 0.01, 0.07; CRP-ADHD: OR_IVW_ = 1.09, 95% CI = 1.01, 1.17).

**Conclusions:**

Further work is necessary to understand the biological pathways linking ADHD genetic liability and CRP and gain insights into understanding how they might contribute in the links between ADHD and later-life adverse physical and mental health outcomes.

## Graphical abstract

**Figure:**
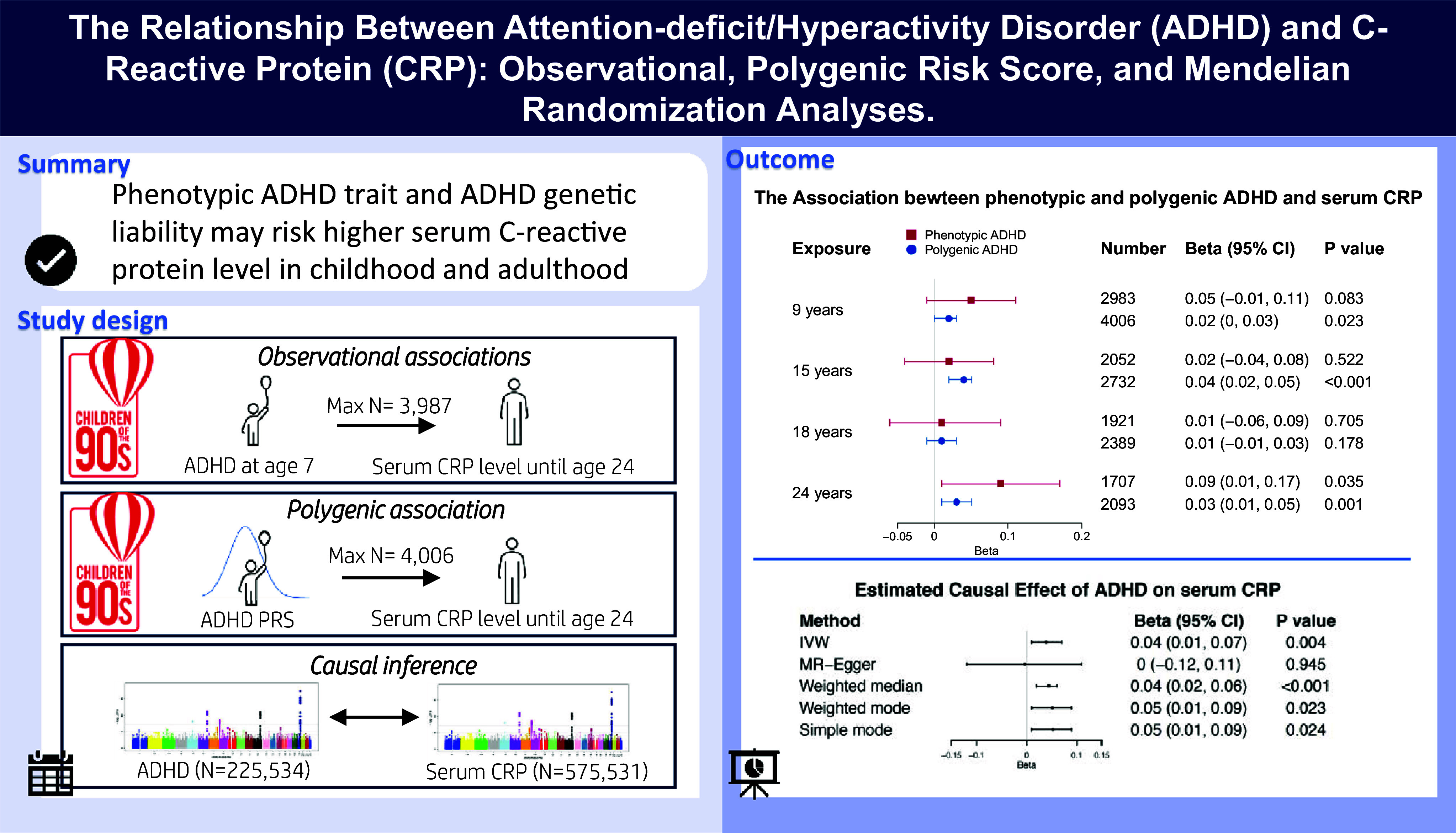

## Introduction

Attention-deficit hyperactivity disorder (ADHD) is a neurodevelopmental condition, typically arising in childhood and characterized by difficulties in behavioral and neurocognitive functioning (Posner, Polanczyk, & Sonuga-Barke, 2020). Individuals with ADHD appear to be at higher risk of a number of adverse physical and mental health outcomes throughout life, particularly cardiovascular conditions, autoimmune conditions, and depression (Dardani et al., 2021; Hegvik et al., 2018; Leppert et al., 2021; Riglin et al., 2021). It has been proposed that a potential mechanism for this could be systemic inflammation (Nigg, 2013). Although there is increasing evidence suggesting that systemic inflammation is a key component of cardiovascular, autoimmune, and a number of mental health conditions (Abou-Raya & Abou-Raya, 2006; Halaris, 2017), its role in ADHD remains less understood. Uncovering whether ADHD is linked to systemic inflammation may have important implications toward improving the health outcomes of the affected individuals.

The association between early childhood ADHD traits and markers of systemic inflammation, such as C-reactive protein (CRP), remains underexplored, particularly within an epidemiological triangulation framework (Lawlor, Tilling, & Smith, 2016). For instance, a cross-sectional study conducted in Taiwan reported higher plasma high-sensitivity CRP in children with ADHD (mean age = 9.32 years) (Chang et al., 2020). In contrast, findings from the 1993 Pelotas Birth Cohort showed little evidence for an observational association between ADHD at 9 years and CRP levels at 18 or 22 years (Leffa et al., 2021). Longitudinal investigations assessing the relationship between ADHD traits and CRP across developmental stages are scarce, and the observational nature of existing research limits the ability to draw conclusions.

The potential mechanisms underlying the links between ADHD and low-grade systemic inflammation are unknown. One possibility is that shared genetic and/or environmental factors may contribute to the complex relationships. For example, the latest genome-wide association study (GWAS) of ADHD found enrichment for some pathways influencing cytokine production (interleukins 1B, 2, 4, 6, and 10), although these findings did not survive multiple testing corrections (Demontis et al., 2023). To better understand the relationships between ADHD and inflammation and gain insights into their underlying mechanisms, it is important to integrate methodologies that combine both observational and polygenic approaches.

This study aims to elucidate the links between ADHD and low-grade systemic inflammation by integrating diverse methodological approaches that leverage both phenotypic and genetic data. Using data from a large population-based cohort, along with publicly available GWAS summary data, we assessed whether: (1a) phenotypic expression of ADHD traits at age 7 years and (1b) common variant genetic liability to ADHD are associated with the levels of CRP from childhood to early adulthood. Furthermore, we performed secondary analyses to assess whether: (2a) there is a shared genetic background between ADHD and CRP, by estimating their genetic correlation and applying linkage-disequilibrium score regression (LDSC); and (2b) there is a causal relationship between the common variant genetic liability to ADHD and biomarkers of systemic inflammation, inlcuding CRP, and cytokines related to immunological biological pathways, using two-sample Mendelian randomization (MR).

## Materials and methods

### Primary investigations: phenotypic and polygenic associations of ADHD with CRP

#### Sample: the Avon Longitudinal Study of Parents and Children (ALSPAC) birth cohort

The ALSPAC, a birth cohort based in the Avon region (UK), was used to examine the relationship between ADHD traits, ADHD genetic liability, and serum CRP levels. Pregnant women who were residents of Avon, UK, with expected dates of delivery between April 1, 1991, and December 31, 1992, were invited to take part in the study. A total of 20,248 pregnancies were identified as being eligible, and the initial number of pregnant women enrolled in the study was 14,541. Of the initial pregnancies, there was a total of 14,676 fetuses, resulting in 14,062 live births, and 13,988 children who were alive at 1 year of age. The total sample size for analyses using any data collected after the age of 7 years is 15,447 pregnancies, resulting in 15,658 fetuses. Of these, 14,901 children were alive at 1 year of age. The details of the cohort have been presented in previous work (Boyd et al., 2013; Fraser et al., 2013). Data on the children and their parents were collected through questionnaire completion, in-person clinic visits, and genotyping. The study website contains details of all the data available through a fully searchable data dictionary: http://www.bristol.ac.uk/alspac/researchers/access/. Consent for biological samples has been collected in accordance with the Human Tissue Act (2004). Informed consent for the use of data collected via questionnaires and clinics was obtained from participants following the recommendations of the ALSPAC Ethics and Law Committee at the time (http://www.bristol.ac.uk/alspac/researchers/research-ethics/).

#### Exposure: ADHD traits at age 7 years

In line with previous studies in the ALSPAC cohort (Riglin et al., 2016; Riglin et al., 2021), we used the hyperactivity subscale (score range 0–10) of the parent-reported Strength and Difficulties Questionnaire (SDQ) (Goodman, 1997), administered when the children were 7 years old. Scores of 7 or higher on this subscale were considered indicative of ADHD.

#### Exposure: common variant genetic liability to ADHD

We constructed polygenic risk scores (PRS) for ADHD in children of the ALSPAC cohort (Collister, Liu, & Clifton, 2022). We used as a discovery sample the latest GWAS meta-analysis of ADHD, with a total sample of 38,691 ADHD cases and 186,843 controls of European ancestry. Details on the GWAS can be found in the original publication (Demontis et al., 2023). Individual-level genotype data from the ALSPAC cohort were used as the target sample. A total of 9,912 children of the ALSPAC cohort were genotyped using the Illumina HumanHap550-quad SNP genotyping platform, with imputation based on the 1,000 Genomes project. After quality control (details in Supplementary Note 1) and removing participants who had withdrawn consent, genotype data were available for 7,857 participants. There was no sample overlap between the discovery data (GWAS for ADHD) and target data (ALSPAC).

PRSs for ADHD were estimated in the eligible sample using PLINK 1.9. Genetic variants from the target sample were excluded if: (a) there were allelic mismatches between the target and discovery samples; (b) the variants were located in the wider major histocompatibility complex region (the genomic region on chromosome 6 spanning from base pair position 25,000,000 to 34,000,000, hg 19 build 37) (Euesden, Lewis, & O’Reilly, 2015). After including only variants with imputation quality score > 0.8 and minor allele frequency > 0.01, the variants were clumped with an *r*^2^ parameter of 0.25 and a physical distance threshold for 500 kB. Palindromic single nucleotide polymorphisms (SNPs) were excluded before the calculation for PRS. PRSs were then estimated using eight *p*-value thresholds for SNP inclusion: 0.5, 0.1, 0.05, 0.005, 0.001, 1 × 10^−5^, 5 × 10^−6^, and 5 × 10^−8^. We considered 0.5 as the primary threshold in line with previous relevant work in the ALSPAC cohort (Riglin et al., 2016). PRSs were standardized by subtracting the mean and dividing by the standard deviation.

#### Outcome: serum CRP levels at ages 9, 15, 18, and 24 years

Serum CRP levels were assessed at four time points. At age 9 years, nonfasting blood samples from the participants were assayed after a median of 7.5 years in storage. Serum CRP measurements were collected from 5,013 children from the ALSPAC cohort and ranged from 0.01 to 67.44 mg/l. At age 15 years, the participants provided blood samples after a fast of at least 6 h. Samples were immediately spun and frozen at -80°C and were assayed after 3–12 months. Similar procedures were followed for measuring CRP at age 18. A total of 3,451 valid serum CRP measurements ranging from 0.07 to 72.55 mg/l and 3,247 valid serum CRP measurements ranging from 0.02 to 176.1 mg/l were obtained for ages 15 and 18, respectively. At age 24 years, CRP was measured by particle-enhanced immunoturbidimetric assay. A total of 2,978 valid serum CRP measurements ranging from 0.1 to 224.72 mg/l were obtained for this time point. All assay coefficients of variation were < 5%.

In line with previous work in the ALSPAC cohort, individuals with CRP levels >10 mg/L were excluded from analysis, as these levels of CRP could potentially indicate the current acute inflammation in pediatric populations, for example, infection (Perry, Zammit, Jones, & Khandaker, 2021). Due to skewed distributions, CRP data were log-transformed for subsequent analyses.

#### Covariates

In analyses investigating the observational associations between ADHD in childhood and serum CRP levels, we considered the following covariates: maternal highest educational qualification, crowding index collected at 18 weeks gestation, and financial difficulties collected at 32 weeks gestation, maternal age at delivery, pre-pregnancy body mass index, maternal depression derived from Edinburgh Postnatal Depression Score (EPDS) collected at 18 weeks gestation, maternal anxiety derived from Crown-Crisp Experiential Index (CCEI) – anxiety phobic anxiety scale, and participant sex and gestational age. Details on the covariates and the rationale for their selection can be found in Supplementary Note 2.

In analyses investigating the polygenic associations between common variant genetic liability to ADHD and serum CRP levels, we used sex (male/female) and the 10 first principal components in ALSPAC as covariates.

#### Statistical analyses

Statistical analyses were conducted in R version 3.6.3. We compared individuals with and without ADHD traits at age 7 years, as well as available genotype data, on covariate data and serum CRP levels, using Pearson *χ^2^*-test, independent-samples *t*-tests, and linear regression analyses. Using linear regression, we estimated regression coefficients and 95% confidence intervals (CIs) for the associations between ADHD traits at age 7 years, as well as common variant genetic liability to ADHD and CRP levels at ages 9, 15, 18, and 24 years. We performed crude and covariate-adjusted analyses. To assess potential sex-specific effects, we conducted analyses stratified by sex when evaluating the association between ADHD traits, as well as ADHD genetic liability and CRP.

### Secondary investigations: genetic correlations and causal relationships between common variant genetic liability to ADHD, CRP, and inflammatory cytokines

#### Genetic correlation analyses: LDSC

We used LDSC (Bulik-Sullivan et al., 2015) to estimate the genetic correlation between ADHD and CRP. LDSC allows the estimation of the genetic correlation between polygenic traits using GWAS summary statistics. We used the latest available GWAS summary data on ADHD (*N*_cases_ = 38,691; *N*_controls_ = 186,843) (Demontis et al., 2023) and CRP (*N* = 575,531) (Said et al., 2022). Details can be found in the original GWAS publication. There was no overlap between the samples used in the GWAS for ADHD and those used in the GWAS for CRP.

We followed the suggested protocol for LDSC analyses (https://github.com/bulik/ldsc/wiki). Using the LDSC (LD score) v.1.0.1 software in Python v.3.9.7, we estimated genetic correlation using precomputed LD scores from the 1000 Genomes project European data (https://data.broadinstitute.org/alkesgroup/LDSCORE/eurwld_chr.tar.bz).

#### Causal inference analyses: two-sample MR

MR is a causal inference approach utilizing genetic variants as instrumental variables to assess the causal effect of an exposure on an outcome (Sanderson et al., 2022; Smith & Hemani, 2014). MR relies on strict assumptions that the instruments should satisfy. Specifically, they must be: (i) strongly associated with the exposure; (ii) independent of any confounders related to both exposure and outcome; and (iii) affect the outcome only via the exposure (Burgess, Butterworth, & Thompson, 2013). The assumptions of two-sample MR are presented in Supplementary Figure S1.

We used two-sample MR to assess the causal effects of genetic liability to ADHD on the levels of plasma CRP as well as the levels of T-helper cell pathway cytokines. Information on T-helper cell pathways and their related cytokines can be found in Supplementary Note 3.

Using the latest publicly available GWAS summary data on ADHD, we extracted genetic instruments for ADHD. Specifically, a total of 26 independent common genetic variants for ADHD were available (*P* < 5 × 10^−8^; *r*^2^ < 0.001; 10,000 kB). For each genetic instrument, we extracted effect sizes and standard errors from the GWAS of the outcome. For each outcome of interest (CRP and levels of T-helper pathway cytokines), we used publicly available GWAS summary data. Details on the CRP GWAS can be found in the LDSC section above. With regard to the GWASs on the levels of T-helper pathway cytokines, we used GWAS data from the INTERVAL study (*N* = 3,301) (Sun et al., 2018). Our primary method of analysis was the inverse variance weighted (IVW) method. We additionally performed sensitivity analyses to test the robustness of the effect estimates: MR-Egger regression (Burgess & Thompson, 2017), weighted median (Bowden, Smith, Haycock, & Burgess, 2016), and weighted mode (Hartwig, Smith, & Bowden, 2017). Details on each method of analysis applied can be found in Supplementary Note 4.

To assess the possibility of reverse causation, the Steiger filtering test was implemented to assess whether the genetic variants used as instruments explained more variance in the outcome than the exposure (Hemani, Tilling, & Smith, 2017). We also performed bidirectional MR analyses investigating the causal effects of genetically proxied serum CRP levels, plasma T-helper cell cytokine levels, and genetically proxied expression of cytokine encoding genes in the brain cortex on ADHD. Details on these analyses, including the genetic instruments used, can be found in Supplementary Note 5. The present MR study followed the Strengthening the Reporting of Observational Studies in Epidemiology using Mendelian Randomization (STROBE MR) guidelines for reporting and the corresponding checklist (Skrivankova et al., 2021).

## Results

### Primary investigations: phenotypic and polygenic associations of ADHD with CRP

The characteristics of participants with and without ADHD at age 7 years can be found in Table 1. Participants with ADHD traits (aged 7 years) were more likely to be male and born preterm compared to those without such traits. The mothers of those with ADHD were more likely to have higher anxiety and depression scores, and conceive at a younger age. In addition, they were more likely to come from less privileged socioeconomic positions, including crowded living conditions, financial difficulties during pregnancy, and have less educational qualifications. The characteristics of participants with and without genotype data can be found in Supplementary Table S1.

**Table 1.** Baseline characteristics in participants with and without ADHD traits assessed by mother-reported SDQ at age 7 years

Variable^a^	Without ADHD traits (*n* = 7391)	With ADHD traits (*n* = 904)	All participants (*n* = 8295)	*P*-value^b^
Sex				
Female	3,736 (50.6)	301 (33.3)	4,037 (48.7)	<0.001
Male	3,643 (49.4)	603 (66.7)	4,246 (51.3)	
Missing	12	0	12	
Gestational age				
Typical	7,018 (95)	836 (92.6)	7,854 (94.7)	0.007
Preterm	341 (4.6)	59 (6.5)	400 (4.8)	
Post term	31 (0.4)	8 (0.9)	39 (0.5)	
Missing	1	1	2	
Maternal age at delivery				
Mean (SD)	28.6 (4.5)	28.1 (4.7)	28.6 (4.6)	<0.001
Missing	181	29	210	
Pre-pregnancy BMI				
Mean (SD)	22.9 (3.7)	23 (4)	22.9 (3.8)	0.588
Missing	632	78	710	
EPDS				
Mean (SD)	6.8 (4.3)	8.3 (4.9)	7 (4.4)	<0.001
Missing	1,101	122	1223	
CCEI-anxiety				
Mean (SD)	12.7 (7.2)	15.7 (8.1)	13 (7.3)	<0.001
Missing	904	126	1030	
Maternal highest educational qualification				
CSE	1,040 (14.4)	160 (18.4)	1,200 (14.9)	<0.001
Vocational	612 (8.5)	101 (11.6)	713 (8.8)	
O level	2,541 (35.3)	313 (36)	2,854 (35.4)	
A level	1,858 (25.8)	197 (22.6)	2,055 (25.5)	
Degree	1,151 (16)	99 (11.4)	1,250 (15.5)	
Missing	189	34	223	
Crowding index				
≤0.5	3,405 (47.8)	370 (42.8)	top3,775 (47.3)	0.001
0.5–0.75	2,239 (31.4)	272 (31.4)	top2,511 (31.4)	
>0.75–1	1,176 (16.5)	168 (19.4)	top1,344 (16.8)	
>1	303 (4.3)	55 (6.4)	358 (4.5)	
Financial difficulties				
Mean (SD)	2.5 (3.3)	3.4 (3.8)	top2.6 (3.3)	<0.001
Missing	353	64	417	

Abbreviations: ADHD, attention-deficit hyperactivity disorder; BMI, body mass index; CCEI, Crown-Crisp Experiential Index; EPDS, Edinburgh Postnatal Depression Scale; SDQ, Strengths and Difficulties Questionnaire; SD, standard deviation.

a A *T*-test was performed for continuous variables and a *χ*^2^-test was performed for categorized variables.

b The *P*-value corresponds to the test for differences between participants with and without ADHD traits at age 7 years.

There was limited evidence to suggest an association between ADHD traits and serum CRP levels from childhood to adulthood. As shown in Table 2, there was some evidence of a positive association between ADHD traits at age 7 years and serum CRP levels at age 24 years, after adjusting for measured confounders (*β* = 0.18, 95% CI = 0.01, 0.34; *P* = 0.034). Little evidence was found for potential sex differences in the associations between ADHD traits and CRP levels across different ages (Supplementary Table S2).

**Table 2.** Associations between ADHD traits assessed by mother-reported SDQ at age 7 years and serum CRP levels in childhood, adolescence, and adulthood

Crude model (univariable regression)				
CRP (years)	ADHD traits	No. participants	*β* (95% CI)	*P*-value
Age 9	Not present	3,606	Reference	
	Present	385	0.02 (−0.1, 0.14)	0.751
Age 15	Not present	2,472	Reference	
	Present	267	0.01 (−0.04, 0.06)	0.716
Age 18	Not present	2,331	Reference	
	Present	209	−0.06 (−0.2, 0.09)	0.45
Age 24	Not present	2,080	Reference	
	Present	182	0.18 (0.01, 0.34)	0.034
Adjusted model (multivariable regression model)^a^				
Age 9	Not present	2,684	Reference	
	Present	302	0.08 (−0.05, 0.21)	0.217
Age 15	Not present	1,853	Reference	
	Present	206	0.01 (−0.13, 0.15)	0.863
Age 18	Not present	1,772	Reference	
	Present	167	0.01 (−0.15, 0.18)	0.865
Age 24	Not present	1,587	Reference	
	Present	137	0.21 (0.03, 0.4)	0.026

Abbreviations: ADHD, attention deficit hyperactivity disorder; BMI, body mass index; CCEI, Crown-Crisp Experiential Index; CRP, C-reactive protein; CI, confidence interval; EPDS, Edinburgh Postnatal Depression Scale; SDQ, Strengths and Difficulties Questionnaire.

a The multivariable model was adjusted for child sex and gestational age, maternal age when pregnant, maternal pre-pregnancy BMI, EPDS, CCEI-anxiety, maternal highest educational qualification, crowding index, and financial difficulties.

In PRS regression analyses, we found evidence of associations between ADHD PRS and serum CRP levels at ages 9 (*β* = 0.02; 95% CI = 0, 0.03; *P* = 0.023), 15 (*β* = 0.04; 95% CI = 0.02, 0.05; *P* < 0.001), and 24 years (*β* = 0.03; 95% CI = 0.01, 0.05; *P* = 0.001) but not at 18 years (*β* = 0.01; 95% CI = −0.01, 0.03; *P* = 0.178; Table 3). Polygenic associations between ADHD and CRP using different SNP-inclusion *P*-value thresholds are presented in Supplementary Table S3. There was little evidence for sex differences in the association between ADHD genetic liability and CRP levels (Supplementary Table S4).

**Table 3.** Associations between common variant genetic liability to ADHD (c, *P* < 0.5) and serum CRP levels in childhood, adolescence, and adulthood

Age (years)	No. participants	*β* (95% CI)^a^^,^^b^^,^^c^	*P*-value
9	4,006	0.02 (0, 0.03)	0.023
15	2,732	0.04 (0.02, 0.05)	<0.001
18	2,389	0.01 (−0.01, 0.03)	0.178
24	2,093	0.03 (0.01, 0.05)	0.001

Abbreviations: ADHD, attention deficit hyperactivity disorder; BMI, body mass index; CCEI, Crown-Crisp Experiential Index; CI, confidence interval; CRP, C-reactive protein; PRS, polygenic risk score.

a All models were additionally adjusted for sex and the first 10 principal components.

b The PRS for ADHD was standardized.

c The serum CRP level was log-transformed to normalize the distribution of CRP.

### Secondary investigations: genetic correlations and potential causal relationships between common variant genetic liability to ADHD, CRP, and inflammatory cytokines

#### Genetic correlation analyses: LDSC

We found a positive genetic correlation (*r*_g_) between ADHD and serum CRP levels (*r*_g_ = 0.27, SE = 0.04, *P* = 5.04 × 10^−11^).

#### Causal inference analyses: two-sample MR

**Figure 1.**
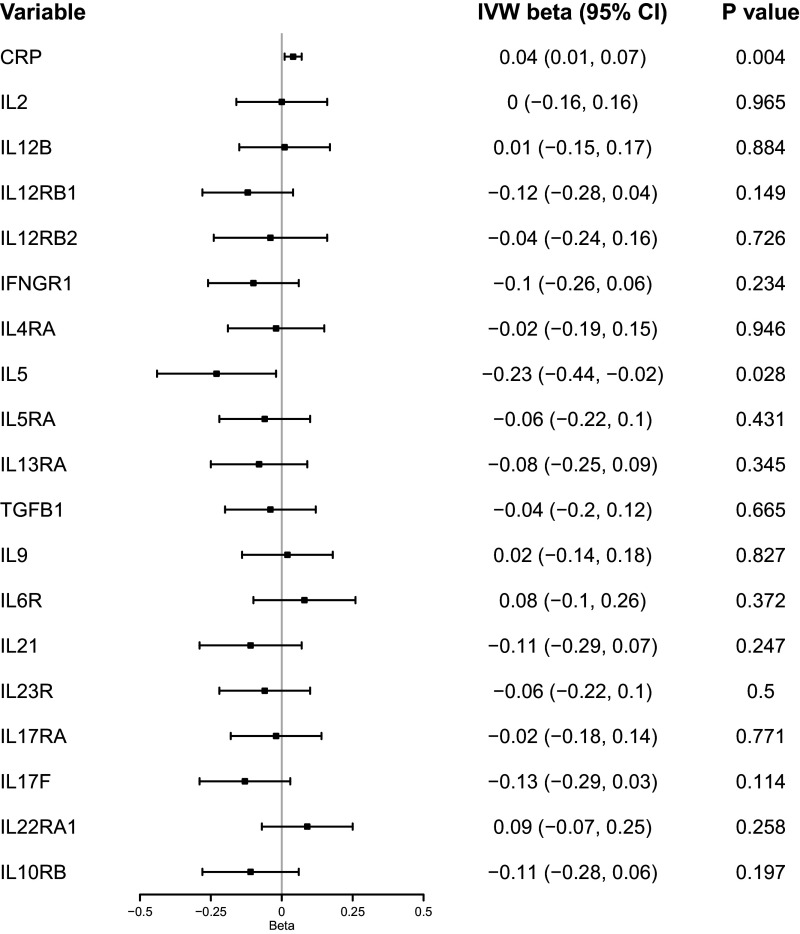
The estimated causal effect of genetic liability to ADHD on plasma cytokines levels derived from two-sample Mendelian randomization. *Note*: ADHD, ‘attention-deficit/hyperactivity disorder’; CRP, ‘C-reactive protein’; IL-2, ‘interleukin 2’; IFNGR1, ‘interferon-γ receptor 1’; IL12B, ‘interleukin-23’; IL12RB1, ‘interleukin-12 receptor subunit β1’; IL12RB2, ‘interleukin-12 receptor subunit β2’; IL4RA, ‘interleukin-4 receptor subunit α’; IL5,’ interleukin-5’; IL5RA, ‘interleukin-5 receptor subunit α’; IL13RA1, ‘interleukin-13 receptor subunit α1’; IL9,’ interleukin-9’; IL6R, ‘interleukin‐6 receptor subunit α’; IL21, ‘interleukin-21’; IL23R, ‘interleukin-23 receptor’; IL17RA, ‘interleukin-17 receptor A’; IL17F, ‘interleukin-17F’; IL22RA1, ‘interleukin-22 receptor subunit α1’; IL10RB, ‘interleukin 10 receptor subunit β’; TGFB1, ‘transforming growth factor β1’.

The F-statistic of the genetic instruments for ADHD ranged from 30 to 60, indicating good instrument strength (Supplementary Table S5). There was evidence to suggest a potential causal effect of genetic liability to ADHD on levels of serum CRP (*β*_IVW_ = 0.039; 95% CI = 0.01, 0.07; *P* = 0.004, Figure 1). The direction of the effect estimates was consistent across the sensitivity analyses, except for the MR-Egger method, while there was limited evidence for horizontal pleiotropy (Intercept_MR-Egger_ = 0.003, *P* = 0.457; see Supplementary Table S6, available online). All the ADHD genetic instruments passed the Steiger filtering test. Detailed results across IVW and sensitivity analyses can be found in Supplementary Table S6.

There was little evidence to suggest an effect of genetic liability to ADHD on the levels of T-helper pathway cytokines (Figure 1). Detailed results across IVW and sensitivity analyses can be found in Supplementary Table S7.

In bidirectional MR analyses, a total of 239 genetic instruments for CRP were included. The F-statistic of the genetic instruments ranged from 29 to 1536, indicating good instrument strength. Details on the genetic instruments for these analyses can be found in Supplementary Tables S8–S10, and proxy SNPs for instrumental variables are presented in Supplementary Table S11. There was evidence to suggest an effect of genetically proxied serum CRP levels on ADHD (OR_IVW_ = 1.09; 95% CI = 1.01, 1.17; *P* = 0.021, Supplementary Figure S2). After excluding SNPs indicated by the Steiger filtering, 234 SNPs remained, and the effect estimates were comparable across analyses (i.e. before and after the exclusion, Supplementary Table S12).

The F-statistic of the genetic instruments ranged from 31 to 2190 for protein levels and from 22 to 737 for gene expression. There was some evidence to suggest an effect of genetically proxied interleukin-4 receptor subunit α (odds ratio [OR] = 1.25; 95% CI = 1.08, 1.46; *P* = 0.004, Supplementary Figure S2) on ADHD. In addition, evidence suggested an effect of genetically proxied interleukin-12 receptor subunit β2 expression in the brain cortex on ADHD (OR = 1.11; 95% CI = 1, 1.23; *P* = 0.049), as well as an effect of genetically proxied interleukin 6 and interleukin 23 subunit α (IL23A) in the brain cortex on ADHD (IL6: OR = 0.86; 95% CI = 0.73, 1; *P* = 0.044; IL23A: OR = 0.91; 95% CI = 0.84, 0.99; *P* = 0.03; Supplementary Figure S2).

## Discussion

In this study, we investigated the associations between ADHD traits in childhood, as well as genetic liability to ADHD and serum CRP levels in childhood, adolescence, and adulthood. We additionally estimated genetic correlation and potential effects of genetic liability to ADHD with CRP, as a marker of systemic inflammation, and T-helper pathway-related cytokines. We found some evidence to indicate associations between ADHD traits in childhood and elevated serum CRP levels in adulthood. We found evidence of associations between ADHD common variant genetic liability and serum CRP levels in childhood, adolescence, and adulthood. Two-sample MR and genetic correlation analyses further supported this evidence by indicating genetic correlations and bidirectional effects between ADHD and CRP. Evidence was less consistent in the case of T-helper pathway cytokines and their potential relationship with ADHD.

Our findings suggest a potential link between ADHD genetic liability and elevated CRP levels. We found evidence suggesting that common variant genetic liability to ADHD is associated with higher serum CRP levels at ages 9, 15, and 24 years, with a weaker but consistent direction of association at age 18 years. The weaker association at age 18 years may reflect random variation, limited statistical power, or developmental processes such as puberty, during which hormonal fluctuations can influence immune activity and the production of inflammatory markers like CRP (Ucciferri & Dunn, 2022). Several mechanistic pathways for our findings could be hypothesised. For example, a potential explanation could be that individuals with higher ADHD genetic liability and therefore likelihood to express the phenotype, may be more likely to experience adverse experiences, such as neglect or physical and emotional abuse, across developmental stages (Stern et al., 2018). These stressors may contribute to increased levels of low-grade systemic inflammation (Kiecolt-Glaser, Gouin, & Hantsoo, 2010).

Another potential explanation for the associations might be shared underlying genetics. The PRS findings, along with the genetic correlation findings and bi-directional MR findings, point to a potential shared genetic basis and may contribute toward understanding the links of ADHD with mental health conditions, such as depression, and autoimmune conditions, such as rheumatoid arthritis. For example, previous work has suggested a central role of systemic inflammation, as reflected by CRP, in depression and rheumatoid arthritis (Osimo et al., 2019; Pope & Choy, 2021). On this basis, it seems important for future work to investigate the interplay between ADHD genetic liability and phenotypic expression, systemic inflammation, and comorbid conditions.

Our study has several strengths. First, we implemented multiple study designs, including cohort, polygenic risk score, genetic correlation, and MR. Second, we used different time points for CRP, enabling an exploration of its relationship with ADHD across both childhood and adulthood.

Our findings should be interpreted in the context of their limitations. One major consideration is that although PRS approaches are less susceptible to bias from unmeasured confounders compared to observational approaches, using a relaxed *P* threshold from GWAS to maximize the variance explained in the phenotype can result in pleiotropic effects (i.e. the included SNPs are also associated with other traits). Similarly, in our bidirectional two-sample MR, sensitivity analyses, including MR-Egger, weighted median, and weighted mode methods, revealed inconsistencies in the estimated effect of genetically proxied levels of CRP on ADHD, with MR-Egger indicating significant pleiotropy. This suggests that the apparent effect of genetically proxied levels of CRP on ADHD may be driven, at least in part, by pleiotropic pathways rather than a direct causal relationship. The presence of pleiotropy highlights the complexity of disentangling the specific role of inflammation in ADHD, as genetic variants influencing CRP levels may also affect ADHD risk through other biological mechanisms. On this basis, the limitations posed by pleiotropy emphasize the importance of cautious interpretation and the need for more robust evidence to establish causality. Second, it is worth noting that our MR and PRS findings reflect a common variant genetic liability to ADHD and, therefore, do not capture all the elements that may lead to the phenotypic expression of ADHD (e.g. rare variation). In addition, although we used a threshold identified previously to maximally capture variance in ADHD, the variance that is currently explained by PRSs in psychiatric phenotypes is still relatively small. Therefore, we recommend caution with regard to potential conclusions on the influences of ADHD genetic liability on systemic inflammation. Third, although the ADHD subscale of the SDQ demonstrates a strong validity in distinguishing DSM-5 ADHD cases from noncases (Riglin et al., 2021), it is important to note that it is not a substitute for a comprehensive clinical assessment and diagnosis of ADHD. Fourth, a large proportion of ALSPAC participants had missing data and were excluded from our analysis. This reduces statistical power and could introduce selection bias if the included individuals are not representative of the whole sample. As a result, the findings should be interpreted cautiously, especially when considering their relevance to the wider population. Future studies in large biobanks, such as the UK Biobank, are expected to further elucidate the identified links between a common variant genetic liability to ADHD and levels of CRP. Fifth, although we excluded serum CRP above 10 ml/L to focus on low-grade inflammation, it may not perfectly differentiate between acute and low-grade inflammation, leading to potentially capturing some cases of acute inflammatory states, for example, active infections. Sixth, we did not correct for multiple testing as the markers assessed are not independent but rather organized into interacting systems. This complicated the determination of the number of independent tests conducted. Instead, we encourage readers to interpret the findings within the context of the consistency of the estimates and their CIs across the analyses conducted (Sterne & Smith, 2001). Finally, the ALSPAC cohort is not representative of the whole UK population, characterized predominantly by socioeconomically advantaged individuals, and very little ethnic diversity (Boyd et al., 2013). Similarly, both PRS analyses using individual-level data and two-sample MR analyses using summary-level data were derived from European ancestry individuals, limiting the interpretation across ancestries. Research using more diverse populations is necessary to better understand the identified relationships in the present study.

## Conclusion

We found evidence suggesting polygenic links between genetic liability to ADHD and a biomarker of systemic inflammation, CRP in childhood, adolescence, and adulthood. Future studies are expected to elucidate the extent to which these findings reflect the influence of shared genetics and/or causal links, as well as the biological pathways implicated. This research avenue is expected to further the current understanding of the health outcomes of ADHD.

## Supporting information

Luo et al. supplementary material 1 Luo et al. supplementary material

Luo et al. supplementary material 2 Luo et al. supplementary material

## Supplementary material

The supplementary material for this article can be found at http://doi.org/10.1017/S0033291725000480.
