# Supplementary material for: Investigating the relationship between attention-deficit hyperactivity disorder (ADHD) and C-reactive protein (CRP): observational, polygenic risk score, and Mendelian randomization analyses
Source: Psychol Med. Author manuscript; Available in PMC 2026 Jul 16. (PMC12094638; doi:10.1017/S0033291725000480)
Supplement: Supplementary Material 1 [file EMS213623-supplement-Supplementary_Material_1.docx]

**Supplementary Notes**

Contents

**Supplementary Note 1: Details on genotyping in ALSPAC**

ALSPAC children were genotyped using the Illumina HumanHap550 quad chip genotyping platforms. The resulting raw genome-wide data were subjected to standard quality control methods. Individuals were excluded on the basis of gender mismatches; minimal or excessive heterozygosity; disproportionate levels of individual missingness (>3%) and insufficient sample replication (IBD < 0.8). Population stratification was assessed by multidimensional scaling analysis and compared with Hapmap II (release 22) European descent (CEU), Han Chinese, Japanese and Yoruba reference populations; all individuals with non-European ancestry were removed. SNPs with a minor allele frequency of < 1%, a call rate of < 95% or evidence for violations of Hardy-Weinberg equilibrium (P < 5*10^-7^) were removed. Cryptic relatedness was measured as a proportion of identity by descent (IBD > 0.1). Related subjects that passed all other quality control thresholds were retained during subsequent phasing and imputation. 9,115 subjects and 500,527 SNPs passed these quality control filters.

After combining genotype data in the mothers and the children, SNPs with genotype missingness above 1% were removed due to poor quality (11,396 SNPs removed) and a further 321 subjects were removed due to potential ID mismatches. This resulted in a dataset of 17,842 subjects. Imputation of the target data was performed using Impute V2.2.2 against the 1000 genomes reference panel (Phase 1, Version 3) (all polymorphic SNPs excluding singletons), using all 2186 reference haplotypes (including non-Europeans).

This gave 8,237 eligible children and 8,196 eligible mothers with available genotype data after the exclusion of related subjects using cryptic relatedness measures described previously.

**Supplementary Note 2: Covariates used in the study investigating the links between ADHD at age 7 and levels of CRP at ages 9, 15, 18 and 24.**

**Covariates (participants):**

**Participants Sex**

Literature evidence: ADHD is more common in boys than girls and appears to have links to different conditions in males vs females (Willcutt, 2012). In the case of CRP, evidence suggests that peripheral levels of CRP might be higher in women compared to men when assessed in healthy participants(Cartier et al., 2009; Lakoski et al., 2006).

In ALSPAC, participants’ sex was collected from the ‘delivery questionnaire’ which was completed from medical records.

**Gestational age:**

Literature evidence: Evidence suggests that preterm birth may be linked to childhood ADHD symptoms (James et al., 2020). In the case of CRP, preterm babies may be more susceptible to adverse immunological events such as infections (Humberg et al., 2020).

In ALSPAC, participants’ gestational age was derived from the baseline data, length of pregnancy (weeks) was collected after birth. Participants with 19-36 weeks gestation ere categorized as ‘Preterm birth’, participants with 37-42 weeks gestation were categorized as ‘Normal’, and participants with length of pregnancy above 42 were categorized as ‘Post-term birth’.

**Covariates (Maternal):**

**Maternal Age at delivery**

Literature evidence: Evidence has shown that parental age may be linked to children's health outcomes. Maternal age has been associated to several offspring outcomes, including phenotypes related to neurodevelopmental and psychiatric conditions (autism spectrum disorder, bipolar disorder) and inflammation (type 1 diabetes mellitus, asthma)(Bergh, Pinborg, & Wennerholm, 2019).

In ASLPAC, maternal age was collected when the mother completed the ‘ALSPAC pregnancy, Birth and Infancy (pregnancy to 12 months)’ questionnaire and treated as a continuous variable.

**EPDS (Post-natal depression at** **18 weeks gestation)**

Literature evidence: Maternal depression has been linked to a number of neurodevelopmental outcomes in the offspring (Nidey et al., 2021). Similarly, in the case of CRP, there is evidence suggesting links between maternal depression during pregnancy and offspring inflammatory states (Plant, Pawlby, Sharp, Zunszain, & Pariante, 2016).

In ALSPAC, maternal EPDS was collected from the questionnaire collected at 18 weeks’ gestation. The EDPS was developed to identify women who may have postpartum depression. Each answer is given a score of 0 to 3. The maximum score is 30. A score of more than 10 suggests minor or major depression may be present.

**CCEI-anxiety (Post-natal anxiety at 18 weeks gestation)**

Literature evidence: Maternal stress during pregnancy has been associated to a number of adverse neurodevelopmental outcomes in the offspring (Bronson & Bale, 2016). Similarly, maternal anxiety has been found to be associated with reduced adaptive immune responses in infants, both humoral and cell-mediated (O’Connor et al., 2013).

In ALSPAC, maternal total Crown Crisp score for anxiety was collected from the questionnaire collected at 18 weeks’ gestation. The CCEI evolved from the Middlesex Hospital Questionnaire and is/was a 48-item measure with scales with six scales, which can be subdivided into six 8-item sub-tests, including anxiety. Anxiety subscale measures the intensity and frequency of anxiety-related experiences, and higher score would suggest that the individual may have significant anxiety symptoms, such as persistent worry, nervousness, or fear.

**Maternal pre-pregnancy BMI**

Literature evidence: Maternal BMI pre-pregnancy may be linked to offspring neurodevelopmental outcomes. For example, a study using data from the Danish National Birth Cohort (DNBC) demonstrated that maternal weight status during pregnancy? Before pregnancy? is linked to an increased risk of ADHD in offspring. Compared to children of typical-weight mothers, those born to overweight mothers had a 28% higher risk of ADHD (HR = 1.28, 95% CI 1.15–1.48). The risk further increased for children of obese mothers, with a 47% higher likelihood (HR = 1.47, 95% CI 1.26–1.71), and was highest among children of severely obese mothers, nearly doubling the risk (HR = 1.95, 95% CI 1.58–2.40) (Andersen, Thomsen, Nohr, & Lemcke, 2018). Similarly in the case of CRP, maternal obesity might impact fetal and offspring neuroinflammation; increased oxidative stress, dysregulated insulin, glucose, and leptin signalling; dysregulated serotonergic and dopaminergic signalling (Edlow, 2017).

In ASLPAC, Mothers were asked, *"What was your weight before you started this pregnancy?"* and *"How tall are you?"* based on a retrospective questionnaire administered at 12 weeks of gestation. Pre-pregnancy BMI was calculated using these two variables. Maternal BMI before pregnancy was calculated by dividing the maternal self-reported pre-pregnancy weight (kg) by the square of height (m).

**Covariates (Socioeconomic position):**

Literature evidence: Research consistently indicates a strong relationship between family socioeconomic status (SES) and the risk of childhood attention-deficit/hyperactivity disorder (ADHD). A study found that children in more chaotic households had higher ADHD symptoms (Agnew-Blais et al., 2022). On the other hand, financial difficulty was suggested as the strongest predictor among several family socioeconomic indicators of ADHD (OR = 2.23 95% CI = 1.57-3.16) (Russell, Ford, & Russell, 2015).

For the association between family environment and chronic inflammation, early-life socioeconomic conditions have a lasting impact on health. Exposure to adversity during childhood can alter immune system development, predisposing individuals to heightened inflammatory responses in adulthood (Cohen, Janicki-Deverts, Chen, & Matthews, 2010). Another study suggested that social adversity (parental education, occupation, and family income) prenatally is associated with elevated CRP in adulthood (mean age = 42.2 years) (Slopen et al., 2015).

**Maternal Highest Educational Qualification**

Mother’s highest educational qualification was collected at 32 weeks’ gestation. Participants reported on having any of the following: 'CSE', 'Vocational', 'O level', 'A level', and 'Degree'.

**Crowding Index**

The crowding index is a measure of how many people live in a household relative to the number of rooms. The index is calculated by dividing the number of people in a household by the number of rooms, excluding bathrooms, toilets, balconies, and kitchens. This variable was further reclassified as ‘<=0.5’, ‘>0.5-0.75’, ‘>0.75-1’, and ‘>1’.

**Financial Difficulties**

The financial difficulties measure was derived from a set of five questions at 32 weeks gestation. Mothers were asked to rate, on a scale from 0 to 3, the current level of difficulty in affording essential items for the child, including food, clothing, heating, rent or mortgage, and other necessities. Higher scores indicated greater financial difficulty.

**Supplementary Note 3:** Cytokines are pleiotropic proteins with a key immune role, driving the differentiation of naïve CD4+ T cells into specific subsets. These subsets are characterized by distinct cytokine products and functions, including T helper 1 (T_H_1), T helper 2 (T_H_2), T helper 9 (T_H_9), T follicular helper (T_FH_), T helper 17 (T_H_17), and regulatory T cells (T_Reg_)(Christina Dardani et al., n.d.).

Table S1. An overview of inductive and product cytokines as well as subset functions.

| CD4^+^ T cell subset | T_H_1 | T_H_2 | T_H_9 | T_FH_ | T_H_17 | T_Reg_ |
| --- | --- | --- | --- | --- | --- | --- |
| Inductive Cytokine | IL-2  IL-12 | IL-2  IL-4 | 1L-2  IL-4  TGF$\beta$ | IL-6  IL-21 | IL-6  IL-21  IL-23  TGF$\beta$ | IL-2  TGF$\beta$ |
| Product Cytokine | IFN-$\gamma$ | IL-4  IL-5  IL-13 | IL-9 | IL-21 | IL-17A  IL-17F  IL-22 | IL-10  TGF$\beta$ |
| Subset functions | Macrophage activation  Inflammatory response against intracellular pathogens | Eosinophil activation  Allergic and autoimmune response  Anti-tumour immune repones | Response in helminth infections  Allergic and autoimmune response  Anti-tumour immune response | B cell activation  Inflammatory response against extracellular pathogens | Neutrophil activation  Inflammation response against extracellular pathogens  Autoimmune response | Regulation of inflammatory response  Regulation of autoimmune response  Suppression of anti-tumour immune response |

**Supplementary Note 4: Mendelian randomization method**

Inverse Variance Weighted (IVW): IVW combines the individual SNP-exposure and SNP-outcome associations using a weighted linear regression approach, where the weights are proportional to the inverse of the variance of the SNP-exposure associations. This method assumes that all genetic variants are valid instrumental variables and that there is no horizontal pleiotropy (i.e., genetic variants affect the outcome only through the exposure of interest) (Davey Smith & Hemani, 2014).

MR-Egger: MR-Egger does not assume that all genetic variants used as instrumental variables are valid. Instead, it allows for the possibility of horizontal pleiotropy by estimating the intercept term, which represents the average pleiotropic effect across all genetic variants. This intercept term is then used to adjust the causal estimate for the presence of pleiotropy. MR-Egger regression provides unbiased estimates of causal effects even in the presence of horizontal pleiotropy, as long as certain assumptions are met. However, it typically requires larger sample sizes to achieve sufficient statistical power compared to other MR methods like IVW. Additionally, MR-Egger regression is less commonly used than IVW due to its increased complexity and potential limitations (Burgess & Thompson, 2017).

The Weighted Median method: a robust approach used to estimate causal effects when there is heterogeneity and potential horizontal pleiotropy among genetic variants used as instrumental variables. The Weighted Median method provides a consistent estimate of the causal effect under certain assumptions, even if up to 50% of the weight in the analysis comes from invalid instruments. It is considered a valuable tool in MR analysis, particularly when there is uncertainty about the presence of horizontal pleiotropy or when there is heterogeneity among genetic instruments (Bowden, Davey Smith, Haycock, & Burgess, 2016).

Weighted Mode method: Similar to the Weighted Median method, the Weighted Mode method provides a consistent estimate of the causal effect under certain assumptions, even when a substantial proportion of the genetic instruments may be invalid. It is useful in MR analysis when there is heterogeneity among genetic instruments and uncertainty about the presence of horizontal pleiotropy (Hartwig, Davey Smith, & Bowden, 2017).

**Supplementary Note 5: Bi-directional MR analyses.**

Instrumental variable for CRP: The GWAS analysis of serum CRP was conducted by combining UK Biobank participants and the Cohort for Heart and Aging Research in Genomic Epidemiology (CHARGE) Consortium (N = 575,531). CRP levels were transformed using natural log, and the details of quality control and analysis were presented in the original GWAS paper (Said et al., 2022). The selection of instrumental variables for serum CRP was conducted in R using an r^2^ parameter of 0.01 and a physical distance threshold for clumping SNPs of 10,000 kB among SNPs which reached genome-wide significance (P value < 5*10^-8^). Proxied SNPs were identified if unavailable in outcome GWAS by using linkage disequilibrium (LD) based on 1000 Genomes samples of European ancestry of phase 3 data (r^2^ > 0.8).

Instrumental variable for plasma T-helper cell cytokine levels: Instruments for pQTL and were derived from previous GWASs (Zheng et al., 2020). Blood plasma pQTL data for 15 of the cytokines of interest were available in four studies: Sun et al, 2018 (N= 3,301)(Sun et al., 2018), Folkersen et al., 2017 (N= 3,394)(Folkersen et al., 2017), Suhre et al., 2017 (N= 1,000)(Suhre et al., 2017), Emilsson et al., 2018 (N= 5,457)(Emilsson et al., 2018). Based on published work, Zheng et al. validated genetic instruments across them in terms of consistency and specificity(Zheng et al., 2020). After removing SNPs and proteins encoded by genes within the human major histocompatibility complex (MHQ) region, independent SNPs were selected using a P-value threshold ≤ 5*10^-8^ and a threshold of r^2^ < 0.001 in the gene region. More details on the validation protocol can be found in the original publication.

Instrumental variable for expression of cytokine encoding genes: Brain cortex eQTL data for the genes that encode the cytokines of interest were derived from the largest meta-analysis of brain-derived eQTL datasets (MetaBrain) (de Klein et al., 2023), selected at a genome-wide significant P-value threshold (P < 5*10-8) and LD-clumped in 10,000kb clumping window with r2 <0.001.

The instrumental variables for other proteomic markers were derived from four previously published papers. Proxied SNPs were identified if unavailable in outcome GWAS by using linkage disequilibrium (LD) based on 1000 Genomes samples of European ancestry of phase 3 data (r^2^ > 0.8).

Supplementary Figure 1: The assumption for two-sample Mendelian Randomization.


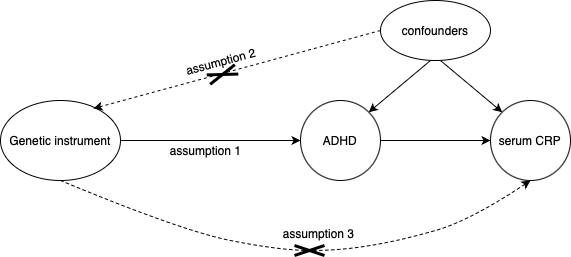


Supplementary Figure 2. The estimated causal effect of inflammation on ADHD derived from two-sample Mendelian randomization.

A. Estimated causal effect of serum CRP on ADHD; B. Estimated causal effect of plasma cytokine levels on ADHD; C. Estimated causal effect of cytokine-encoding gene expression in the brain cortex on ADHD.

IL4RA, IL5RA, IL9, IL17RA, IL17F, AND IL22RA1 in plasma (pQTL) and gene expression for IL5RA and TGFB1 derived from brain cortex (eQTL) were not presented in the table as no ADHD-instrument for exposure can be found in published GWAS for ADHD.

**Reference**

Agnew-Blais, J. C., Wertz, J., Arseneault, L., Belsky, D. W., Danese, A., Pingault, J.-B., … Moffitt, T. E. (2022). Mother’s and children’s ADHD genetic risk, household chaos and children’s ADHD symptoms: A gene–environment correlation study. *Journal of Child Psychology and Psychiatry*, *63*(10), 1153–1163. doi: https://doi.org/10.1111/jcpp.13659

Andersen, C. H., Thomsen, P. H., Nohr, E. A., & Lemcke, S. (2018). Maternal body mass index before pregnancy as a risk factor for ADHD and autism in children. *European Child & Adolescent Psychiatry*, *27*(2), 139–148. doi: 10.1007/s00787-017-1027-6

Bergh, C., Pinborg, A., & Wennerholm, U.-B. (2019). Parental age and child outcomes. *Fertility and Sterility*, *111*(6), 1036–1046. doi: https://doi.org/10.1016/j.fertnstert.2019.04.026

Bowden, J., Davey Smith, G., Haycock, P. C., & Burgess, S. (2016). Consistent Estimation in Mendelian Randomization with Some Invalid Instruments Using a Weighted Median Estimator. *Genetic Epidemiology*, *40*(4), 304–314. doi: 10.1002/gepi.21965

Bronson, S. L., & Bale, T. L. (2016). The Placenta as a Mediator of Stress Effects on Neurodevelopmental Reprogramming. *Neuropsychopharmacology*, *41*(1), 207–218. doi: 10.1038/npp.2015.231

Burgess, S., & Thompson, S. G. (2017). Interpreting findings from Mendelian randomization using the MR-Egger method. *European Journal of Epidemiology*, *32*(5), 377–389. doi: 10.1007/s10654-017-0255-x

Cartier, A., Côté, M., Lemieux, I., Pérusse, L., Tremblay, A., Bouchard, C., & Després, J.-P. (2009). Sex differences in inflammatory markers: what is the contribution of visceral adiposity?2. *The American Journal of Clinical Nutrition*, *89*(5), 1307–1314. doi: https://doi.org/10.3945/ajcn.2008.27030

Christina Dardani, A., Robinson, J. W., Zheng, J., Sadik, A., Pagoni, P., Stergiakouli, E., … affiliations, A. (n.d.). *Immunological pathways underlying autism: Findings from Mendelian randomization and genetic colocalisation analyses*. *10*, 12. doi: 10.1101/2022.02.16.22271031

Cohen, S., Janicki-Deverts, D., Chen, E., & Matthews, K. A. (2010). Childhood socioeconomic status and adult health. *Annals of the New York Academy of Sciences*, *1186*(1), 37–55. doi: https://doi.org/10.1111/j.1749-6632.2009.05334.x

Davey Smith, G., & Hemani, G. (2014). Mendelian randomization: genetic anchors for causal inference in epidemiological studies. *Human Molecular Genetics*, *23*(R1), R89–R98. doi: 10.1093/hmg/ddu328

de Klein, N., Tsai, E. A., Vochteloo, M., Baird, D., Huang, Y., Chen, C. Y., … Westra, H. J. (2023). Brain expression quantitative trait locus and network analyses reveal downstream effects and putative drivers for brain-related diseases. *Nature Genetics*. doi: 10.1038/s41588-023-01300-6

Edlow, A. G. (2017). Maternal obesity and neurodevelopmental and psychiatric disorders in offspring. *Prenatal Diagnosis*, *37*(1), 95–110. doi: 10.1002/pd.4932

Emilsson, V., Ilkov, M., Lamb, J. R., Finkel, N., Gudmundsson, E. F., Pitts, R., … Gudnason, V. (2018). Co-regulatory networks of human serum proteins link genetics to disease. *Science*, *361*(6404). doi: 10.1126/science.aaq1327

Folkersen, L., Fauman, E., Sabater-Lleal, M., Strawbridge, R. J., Frånberg, M., Sennblad, B., … Mälarstig, A. (2017). Mapping of 79 loci for 83 plasma protein biomarkers in cardiovascular disease. *PLoS Genetics*, *13*(4). doi: 10.1371/journal.pgen.1006706

Hartwig, F. P., Davey Smith, G., & Bowden, J. (2017). Robust inference in summary data Mendelian randomization via the zero modal pleiotropy assumption. *International Journal of Epidemiology*, *46*(6), 1985–1998. doi: 10.1093/ije/dyx102

Humberg, A., Fortmann, I., Siller, B., Kopp, M. V., Herting, E., Göpel, W., … German Neonatal Network, G. C. for L. R. and P. I. at the beginning of life (PRIMAL) C. (2020). Preterm birth and sustained inflammation: consequences for the neonate. *Seminars in Immunopathology*, *42*(4), 451–468. doi: 10.1007/s00281-020-00803-2

James, S.-N., Rommel, A.-S., Rijsdijk, F., Michelini, G., McLoughlin, G., Brandeis, D., … Kuntsi, J. (2020). Is association of preterm birth with cognitive-neurophysiological impairments and ADHD symptoms consistent with a causal inference or due to familial confounds? *Psychological Medicine*, *50*(8), 1278–1284. doi: DOI: 10.1017/S0033291719001211

Lakoski, S. G., Cushman, M., Criqui, M., Rundek, T., Blumenthal, R. S., D’Agostino, R. B., & Herrington, D. M. (2006). Gender and C-reactive protein: Data from the Multiethnic Study of Atherosclerosis (MESA) cohort. *American Heart Journal*, *152*(3), 593–598. doi: https://doi.org/10.1016/j.ahj.2006.02.015

Nidey, N. L., Momany, A. M., Strathearn, L., Carter, K. D., Wehby, G. L., Bao, W., … Ryckman, K. (2021). Association between perinatal depression and risk of attention deficit hyperactivity disorder among children: a retrospective cohort study. *Annals of Epidemiology*, *63*, 1–6. doi: https://doi.org/10.1016/j.annepidem.2021.06.005

O’Connor, T. G., Winter, M. A., Hunn, J., Carnahan, J., Pressman, E. K., Glover, V., … Caserta, M. T. (2013). Prenatal maternal anxiety predicts reduced adaptive immunity in infants. *Brain, Behavior, and Immunity*, *32*, 21–28. doi: https://doi.org/10.1016/j.bbi.2013.02.002

Plant, D. T., Pawlby, S., Sharp, D., Zunszain, P. A., & Pariante, C. M. (2016). Prenatal maternal depression is associated with offspring inflammation at 25 years: a prospective longitudinal cohort study. *Translational Psychiatry*, *6*(11), e936–e936. doi: 10.1038/tp.2015.155

Russell, A. E., Ford, T., & Russell, G. (2015). Socioeconomic Associations with ADHD: Findings from a Mediation Analysis. *PLOS ONE*, *10*(6), e0128248-. Retrieved from https://doi.org/10.1371/journal.pone.0128248

Said, S., Pazoki, R., Karhunen, V., Võsa, U., Ligthart, S., Bodinier, B., … Dehghan, A. (2022). Genetic analysis of over half a million people characterises C-reactive protein loci. *Nature Communications*, *13*(1). doi: 10.1038/s41467-022-29650-5

Slopen, N., Loucks, E. B., Appleton, A. A., Kawachi, I., Kubzansky, L. D., Non, A. L., … Gilman, S. E. (2015). Early origins of inflammation: An examination of prenatal and childhood social adversity in a prospective cohort study. *Psychoneuroendocrinology*, *51*, 403–413. doi: https://doi.org/10.1016/j.psyneuen.2014.10.016

Suhre, K., Arnold, M., Bhagwat, A. M., Cotton, R. J., Engelke, R., Raffler, J., … Graumann, J. (2017). Connecting genetic risk to disease end points through the human blood plasma proteome. *Nature Communications*, *8*. doi: 10.1038/ncomms14357

Sun, B. B., Maranville, J. C., Peters, J. E., Stacey, D., Staley, J. R., Blackshaw, J., … Butterworth, A. S. (2018). Genomic atlas of the human plasma proteome. *Nature*, *558*(7708), 73–79. doi: 10.1038/s41586-018-0175-2

Willcutt, E. G. (2012). The Prevalence of DSM-IV Attention-Deficit/Hyperactivity Disorder: A Meta-Analytic Review. *Neurotherapeutics*, *9*(3), 490–499. doi: 10.1007/s13311-012-0135-8

Zheng, J., Haberland, V., Baird, D., Walker, V., Haycock, P. C., Hurle, M. R., … Gaunt, T. R. (2020). Phenome-wide Mendelian randomization mapping the influence of the plasma proteome on complex diseases. *Nature Genetics*, *52*(10), 1122–1131. doi: 10.1038/s41588-020-0682-6
