# Supplementary material for: Investigating the relationship between attention-deficit hyperactivity disorder (ADHD) and C-reactive protein (CRP): observational, polygenic risk score, and Mendelian randomization analyses
Source: Psychol Med. Author manuscript; Available in PMC 2026 Jul 16. (PMC12094638; doi:10.1017/S0033291725000480)
Supplement: Supplementary Material 2 [file EMS213623-supplement-Supplementary_Material_2.docx]

Table S1. The characteristics of participants with and without genetic data.

| Variable | Without genetic data (n=7598) | With genetic data (n=7844) | All participants (n = 15442) | P value^1^ |
| --- | --- | --- | --- | --- |
| hyperactive symptoms at 7 years old | |  |  | 0.1496 |
| No | 2231(91.2) | 5091(92.2) | 7322(91.9) |  |
| Yes | 215(8.8) | 431(7.8) | 646(8.1) |  |
| Missing | 5152 | 2322 | 7474 |  |
| sex |  |  |  | 0.6116 |
| Female | 3437(49.2) | 3822(48.7) | 7259(48.9) |  |
| Male | 3555(50.8) | 4022(51.3) | 7577(51.1) |  |
| Missing | 606 | 0 | 606 |  |
| Gestational age |  |  |  | <0.001 |
| Normal | 5921(92.2) | 7069(94.5) | 12990(93.4) |  |
| Preterm | 479(7.5) | 371(5) | 850(6.1) |  |
| Post term | 23(0.4) | 41(0.5) | 64(0.5) |  |
| Missing | 1175 | 363 | 1538 |  |
| Maternal age when pregnant | |  |  | <0.0001 |
| Mean (SD) | 27(5.1) | 28.4(4.7) | 27.8(4.9) |  |
| Missing | 1456 | 637 | 2093 |  |
| EPDS |  |  |  | <0.0001 |
| Mean (SD) | 7.9(4.9) | 7.1(4.5) | 7.4(4.7) |  |
| Missing | 2601 | 1578 | 4179 |  |
| CCEI-anxiety |  |  |  | <0.0001 |
| Mean (SD) | 5.2(3.7) | 4.7(3.5) | 4.9(3.6) |  |
| Missing | 2454 | 1215 | 3669 |  |
| Maternal BMI before pregnancy |  |  |  | 0.883 |
| Mean (SD) | 22.9(3.9) | 22.9(3.8) | 22.9(3.9) |  |
| Missing | 2678 | 1227 | 3905 |  |
| Maternal education |  |  |  | <0.001 |
| CSE | 1393(26.5) | 1098(15.6) | 2491(20.2) |  |
| Vocational | 589(11.2) | 626(8.9) | 1215(9.9) |  |
| O level | 1811(34.4) | 2451(34.8) | 4262(34.6) |  |
| A level | 999(19) | 1762(25) | 2761(22.4) |  |
| Degree | 470(8.9) | 1113(15.8) | 1583(12.9) |  |
| Crowding index |  |  |  | <0.001 |
| <=0.5 | 2139(35.9) | 3276(46) | 5415(41.4) |  |
| 0.5-0.75 | 1882(31.6) | 2217(31.1) | 4099(31.4) |  |
| >0.75-1 | 1370(23) | 1276(17.9) | 2646(20.2) |  |
| >1 | 563(9.5) | 349(4.9) | 912(7) |  |
| Missing | 1644 | 726 | 2370 |  |
| Financial difficulties |  |  |  | <0.001 |
| Mean (SD) | 3.3(3.7) | 2.6(3.4) | 2.9(3.5) |  |
| Missing | 2486 | 972 | 3458 |  |

^1^ The P value is corresponding to the test for difference between participants with and without genetic data.

SD: Standard deviation; EPDS: Edinburgh postnatal depression scale. CCEI: Crown-Crisp Experiential Index.

Table S2. The association between ADHD symptoms (mother-reported, derived by SDQ) at age 7 and serum CRP level across different age stratified by sex.

|  | ADHD symptoms | N | Beta | P value | P for interaction |
| --- | --- | --- | --- | --- | --- |
| CRP at age 9 | | | | | |
| Male | No | 1341 | Reference |  | 0.818 |
|  | Yes | 195 | 0.1(-0.06,0.26) | 0.237 |  |
| Female | No | 1343 | Reference |  |  |
|  | Yes | 107 | 0.03(-0.19,0.25) | 0.793 |  |
| CRP at age 15 | | | | | |
| Male | No | 898 | Reference |  | 0.152 |
|  | Yes | 132 | -0.08(-0.26,0.09) | 0.35 |  |
| Female | No | 955 | Reference |  |  |
|  | Yes | 74 | 0.14(-0.09,0.37) | 0.226 |  |
| CRP at age 18 | | | | | |
| Male | No | 881 | Reference |  | 0.77 |
|  | Yes | 115 | 0.06(-0.13,0.25) | 0.55 |  |
| Female | No | 891 | Reference |  |  |
|  | Yes | 52 | -0.05(-0.36,0.25) | 0.726 |  |
| CRP at age 24 | | | | | |
| Male | No | 652 | Reference |  | 0.8 |
|  | Yes | 80 | 0.25(0.01,0.49) | 0.045 |  |
| Female | No | 935 | Reference |  |  |
|  | Yes | 57 | 0.16(-0.13,0.45) | 0.266 |  |

Models were adjusted for child gestational age, maternal age when pregnant, maternal pre-pregnancy BMI, EPDS, CCEI-anxiety, maternal education level, crowding index, and financial difficulties.

Table S3. The association between genetic liability to ADHD and serum CRP at different time points (exclude participants with serum CRP >10).

| Threshold | CRP at age 9 | | CRP at age 15 | | CRP at age 18 | | CRP at age 24 | |
| --- | --- | --- | --- | --- | --- | --- | --- | --- |
|  | N = 4006 | | N = 2732 | | N = 2389 | | N = 2093 | |
|  | Beta | P value | Beta | P value | Beta | P value | Beta | P value |
| P<0.5 (No. SNP = 210251) | 0.02 (0-0.03) | 0.023 | 0.04 (0.02-0.05) | <0.001 | 0.01 (-0.01-0.03) | 0.178 | 0.03 (0.01-0.05) | 0.001 |
| P<0.1 (No. SNP = 71586) | 0.01 (0, 0.03) | 0.059 | 0.04 (0.02, 0.06) | <0.001 | 0.02 (0, 0.04) | 0.061 | 0.04 (0.02, 0.06) | 0.001 |
| P<0.05 (No. SNP = 43921) | 0.01 (0, 0.03) | 0.049 | 0.04 (0.02, 0.06) | <0.001 | 0.02 (0, 0.03) | 0.062 | 0.03 (0.01, 0.05) | 0.004 |
| P<0.005 (No. SNP = 9005) | 0.01 (0, 0.03) | 0.054 | 0.04 (0.02, 0.06) | <0.001 | 0.01 (-0.01, 0.03) | 0.281 | 0.03 (0.01, 0.05) | 0.004 |
| P<0.001 (No. SNP = 3123) | 0.01 (0, 0.03) | 0.18 | 0.03 (0.02, 0.05) | <0.001 | 0.01 (-0.01, 0.03) | 0.171 | 0.03 (0.01, 0.05) | 0.003 |
| P<0.00001 (No. SNP = 275) | 0 (-0.01, 0.02) | 0.662 | 0.01 (0, 0.03) | 0.127 | 0.01 (-0.01, 0.02) | 0.5 | 0 (-0.02, 0.02) | 0.918 |
| P<0.000005 (No. SNP = 201) | 0.01 (-0.01, 0.02) | 0.506 | 0.02 (0, 0.04) | 0.02 | 0.01 (-0.01, 0.03) | 0.277 | 0 (-0.02, 0.02) | 0.776 |
| P<0.00000005 (No. SNP =39) | 0 (-0.01, 0.02) | 0.675 | 0 (-0.02, 0.02) | 0.993 | 0 (-0.01, 0.02) | 0.718 | -0.02 (-0.04, 0) | 0.121 |

All models were adjusted for sex and first 10 PC.

Table S4. The association between genetic liability to ADHD (P<0.5) and serum CRP across different age stratified by sex.

|  | N | Beta | P | P for interaction |
| --- | --- | --- | --- | --- |
| CRP at age 9 |  |  |  |  |
| Male | 1699 | 1.04 (0.99, 1.1) | 0.1 | 0.7 |
| Female | 1593 | 1.06 (1, 1.12) | 0.033 |  |
| CRP at age 15 |  |  |  |  |
| Male | 1101 | 1.08 (1.01, 1.14) | 0.014 | 0.482 |
| Female | 1163 | 1.11 (1.05, 1.17 | <0.001 |  |
| CRP at age 18 |  |  |  |  |
| Male | 1001 | 1.05 (0.99, 1.12) | 0.109 | 0.54 |
| Female | 1003 | 1.02 (0.96, 1.1) | 0.471 |  |
| CRP at age 24 |  |  |  |  |
| Male | 748 | 1.08 (1, 1.16) | 0.055 | 0.783 |
| Female | 1007 | 1.09 (1.02, 1.17) | 0.008 |  |

All models were adjusted for sex and first 10 PCs.

Table S5. Genetic instruments used for MR analysis investigating the causal effect of ADHD on inflammation.

| CHR | SNP | POS | Effect allele | Other allele | INFO | SE | P value | Beta | EAF | F-statistic |
| --- | --- | --- | --- | --- | --- | --- | --- | --- | --- | --- |
| 16 | rs1162202 | 61966703 | C | T | 0.968 | 0.0102 | 1.922E-09 | 0.06139609 | 0.61011727 | 36.2310624 |
| 20 | rs6082363 | 21250843 | T | C | 0.999 | 0.0101 | 4.375E-12 | 0.07030002 | 0.29185776 | 48.4471362 |
| 1 | rs549845 | 44076469 | G | A | 0.999 | 0.0102 | 9.033E-15 | 0.07880194 | 0.32514224 | 59.6861349 |
| 8 | rs4925811 | 145802447 | T | G | 0.963 | 0.0101 | 8.303E-09 | -0.0579999 | 0.52825515 | 32.9770958 |
| 3 | rs2886697 | 20724204 | G | A | 0.999 | 0.0096 | 7.898E-10 | 0.05879707 | 0.64145602 | 37.5118869 |
| 10 | rs11255890 | 8784773 | C | A | 0.996 | 0.0097 | 4.144E-08 | 0.05300034 | 0.39894137 | 29.8547739 |
| 4 | rs6537401 | 147099654 | G | A | 0.995 | 0.01 | 1.395E-08 | -0.0568032 | 0.65585776 | 32.2660158 |
| 11 | rs2582895 | 28602173 | C | A | 1 | 0.0096 | 4.094E-14 | 0.07249739 | 0.62074485 | 57.0298565 |
| 4 | rs17576773 | 112217523 | C | T | 0.999 | 0.0151 | 1.625E-10 | 0.0963006 | 0.88137242 | 40.6728004 |
| 3 | rs114142727 | 87015142 | C | G | 0.982 | 0.0403 | 5.126E-10 | 0.25060306 | 0.988 | 38.6689751 |
| 3 | rs115111850 | 43651029 | A | G | 0.999 | 0.0204 | 1.707E-08 | -0.1148947 | 0.94979913 | 31.7204791 |
| 3 | rs17718444 | 71499401 | C | T | 0.996 | 0.0103 | 2.873E-09 | 0.06119857 | 0.66600435 | 35.3027198 |
| 2 | rs1438898 | 145714354 | A | C | 0.999 | 0.0108 | 4.877E-09 | 0.06289968 | 0.76779913 | 33.9194926 |
| 12 | rs704061 | 89771903 | T | C | 1 | 0.0094 | 2.299E-09 | -0.0559039 | 0.55897068 | 35.369475 |
| 10 | rs11596214 | 106453832 | G | A | 0.997 | 0.0095 | 3.167E-08 | 0.05280116 | 0.57380348 | 30.8915477 |
| 6 | rs2025286 | 70858701 | A | C | 0.999 | 0.0093 | 3.997E-09 | -0.0548998 | 0.55051466 | 34.8478083 |
| 5 | rs77960 | 103964585 | G | A | 0.996 | 0.01 | 2.462E-13 | -0.0732053 | 0.6790836 | 53.5901635 |
| 14 | rs76284431 | 98690923 | T | A | 0.998 | 0.0134 | 1.194E-09 | -0.0816006 | 0.84285776 | 37.0831802 |
| 3 | rs2311059 | 51884072 | G | A | 0.981 | 0.0105 | 3.163E-08 | -0.0580953 | 0.30902932 | 30.6128488 |
| 5 | rs10875612 | 144474779 | C | T | 0.998 | 0.0093 | 5.624E-09 | -0.0542978 | 0.47223019 | 34.0877726 |
| 8 | rs7844069 | 93277087 | T | G | 0.991 | 0.0095 | 6.736E-09 | 0.05519816 | 0.40397503 | 33.7599659 |
| 18 | rs76857496 | 5871800 | C | A | 0.988 | 0.014 | 1.242E-08 | 0.08000271 | 0.86088708 | 32.655271 |
| 5 | rs4916723 | 87854395 | A | C | 0.957 | 0.011 | 9.477E-15 | -0.0852965 | 0.56956894 | 60.1280189 |
| 7 | rs9969232 | 114158954 | G | A | 0.997 | 0.01 | 9.983E-12 | -0.0683003 | 0.37548099 | 46.6492473 |
| 7 | rs73145587 | 67685754 | A | T | 0.984 | 0.0184 | 3.665E-08 | 0.10130129 | 0.90254398 | 30.310583 |
| 18 | rs7506904 | 50625779 | G | A | 1 | 0.0098 | 1.242E-08 | -0.0559039 | 0.36702497 | 32.5410955 |

Table S6. The estimated causal effect of ADHD on inflammation conditioning on different assumptions.

| Exposure | Outcome | Method | No.SNP | Beta for intercept | SE for intercept | P for intercept | Beta | SE | Beta (95% CI) | P value |
| --- | --- | --- | --- | --- | --- | --- | --- | --- | --- | --- |
| ADHD | CRP | MR Egger | 26 | 0.003 | 0.004 | 0.4573 | -0.0041 | 0.0591 | 0 (-0.12, 0.12) | 0.9452 |
| ADHD | CRP | Weighted median | 26 | / | / | / | 0.0443 | 0.0116 | 0.06 (0.04, 0.08) | 0.0001 |
| ADHD | CRP | Weighted mode | 26 | / | / | / | 0.0510 | 0.0212 | 0.05 (0.01, 0.09) | 0.0236 |
| ADHD | CRP | Inverse variance weighted | 26 | / | / | / | 0.0393 | 0.0136 | 0.04 (0.01, 0.07) | 0.0038 |

Table S7. Sensitivity analysis for causal effect of ADHD on CRP and plasma cytokines.

Table S7A. Heterogeneity test for causal effect of ADHD on CRP and plasma cytokines.

| exposure | outcome | method | SNPs | beta | se | beta (95% CI) | P value |
| --- | --- | --- | --- | --- | --- | --- | --- |
| ADHD | IL2 | MR Egger | 26 | 0.121 | 0.3512 | 0.12 (-0.57, 0.81) | 0.7334 |
| ADHD | IL2 | Weighted median | 26 | -0.0293 | 0.1123 | -0.03 (-0.25, 0.19) | 0.7942 |
| ADHD | IL2 | Weighted mode | 26 | -0.0084 | 0.1966 | -0.01 (-0.4, 0.38) | 0.9662 |
| ADHD | IL12B | MR Egger | 26 | 0.0656 | 0.3711 | 0.07 (-0.66, 0.8) | 0.8611 |
| ADHD | IL12B | Weighted median | 26 | -0.1167 | 0.1135 | -0.12 (-0.34, 0.1) | 0.3038 |
| ADHD | IL12B | Weighted mode | 26 | -0.0107 | 0.0809 | -0.01 (-0.17, 0.15) | 0.8946 |
| ADHD | IL12RB1 | MR Egger | 26 | 0.2566 | 0.3432 | 0.26 (-0.41, 0.93) | 0.462 |
| ADHD | IL12RB1 | Weighted median | 26 | -0.1472 | 0.1139 | -0.15 (-0.37, 0.07) | 0.1963 |
| ADHD | IL12RB1 | Weighted mode | 26 | -0.305 | 0.2042 | -0.3 (-0.7, 0.1) | 0.1478 |
| ADHD | IL12RB2 | MR Egger | 26 | 0.2448 | 0.4266 | 0.24 (-0.6, 1.08) | 0.5714 |
| ADHD | IL12RB2 | Weighted median | 26 | -0.0238 | 0.1160 | -0.02 (-0.25, 0.21) | 0.8375 |
| ADHD | IL12RB2 | Weighted mode | 26 | -0.0358 | 0.1941 | -0.04 (-0.42, 0.34) | 0.8552 |
| ADHD | IFNGR1 | MR Egger | 26 | 0.2683 | 0.3432 | 0.27 (-0.4, 0.94) | 0.4421 |
| ADHD | IFNGR1 | Weighted median | 26 | 0.0005 | 0.1122 | 0 (-0.22, 0.22) | 0.9962 |
| ADHD | IFNGR1 | Weighted mode | 26 | 0.0504 | 0.1994 | 0.05 (-0.34, 0.44) | 0.8027 |
| ADHD | IL4RA | MR Egger | 26 | -0.2083 | 0.3719 | -0.21 (-0.94, 0.52) | 0.5805 |
| ADHD | IL4RA | Weighted median | 26 | -0.0103 | 0.1152 | -0.01 (-0.24, 0.22) | 0.9287 |
| ADHD | IL4RA | Weighted mode | 26 | -0.0119 | 0.1974 | -0.01 (-0.4, 0.38) | 0.9524 |
| ADHD | IL5 | MR Egger | 26 | 0.1523 | 0.4507 | 0.15 (-0.73, 1.03) | 0.7383 |
| ADHD | IL5 | Weighted median | 26 | -0.2712 | 0.1228 | -0.27 (-0.51, -0.03) | 0.0272 |
| ADHD | IL5 | Weighted mode | 26 | -0.3012 | 0.2212 | -0.3 (-0.73, 0.13) | 0.1855 |
| ADHD | IL5RA | MR Egger | 26 | 0.5376 | 0.3433 | 0.54 (-0.13, 1.21) | 0.1304 |
| ADHD | IL5RA | Weighted median | 26 | -0.0694 | 0.1123 | -0.07 (-0.29, 0.15) | 0.5366 |
| ADHD | IL5RA | Weighted mode | 26 | -0.0189 | 0.1932 | -0.02 (-0.4, 0.36) | 0.9227 |
| ADHD | IL13RA | MR Egger | 26 | -0.0859 | 0.382 | -0.09 (-0.84, 0.66) | 0.8239 |
| ADHD | IL13RA | Weighted median | 26 | 0.0156 | 0.1168 | 0.02 (-0.21, 0.25) | 0.8936 |
| ADHD | IL13RA | Weighted mode | 26 | 0.0592 | 0.2088 | 0.06 (-0.35, 0.47) | 0.779 |
| ADHD | TGFB1 | MR Egger | 26 | 0.3840 | 0.3432 | 0.38 (-0.29, 1.05) | 0.2742 |
| ADHD | TGFB1 | Weighted median | 26 | -0.0518 | 0.1141 | -0.05 (-0.27, 0.17) | 0.6496 |
| ADHD | TGFB1 | Weighted mode | 26 | -0.0535 | 0.1961 | -0.05 (-0.43, 0.33) | 0.7872 |
| ADHD | IL9 | MR Egger | 26 | 0.6139 | 0.3432 | 0.61 (-0.06, 1.28) | 0.0863 |
| ADHD | IL9 | Weighted median | 26 | 0.0744 | 0.111 | 0.07 (-0.15, 0.29) | 0.503 |
| ADHD | IL9 | Weighted mode | 26 | 0.0807 | 0.2337 | 0.08 (-0.38, 0.54) | 0.7327 |
| ADHD | IL6R | MR Egger | 26 | 0.3438 | 0.3892 | 0.34 (-0.42, 1.1) | 0.3858 |
| ADHD | IL6R | Weighted median | 26 | 0.1277 | 0.1126 | 0.13 (-0.09, 0.35) | 0.2568 |
| ADHD | IL6R | Weighted mode | 26 | 0.1639 | 0.2203 | 0.16 (-0.27, 0.59) | 0.4636 |
| ADHD | IL21 | MR Egger | 26 | -0.6034 | 0.3886 | -0.6 (-1.36, 0.16) | 0.1336 |
| ADHD | IL21 | Weighted median | 26 | -0.2349 | 0.1179 | -0.23 (-0.46, 0) | 0.0463 |
| ADHD | IL21 | Weighted mode | 26 | -0.3812 | 0.2384 | -0.38 (-0.85, 0.09) | 0.1223 |
| ADHD | IL23R | MR Egger | 26 | 0.353 | 0.3434 | 0.35 (-0.32, 1.02) | 0.3141 |
| ADHD | IL23R | Weighted median | 26 | 0.0458 | 0.1149 | 0.05 (-0.18, 0.28) | 0.6901 |
| ADHD | IL23R | Weighted mode | 26 | 0.1304 | 0.2089 | 0.13 (-0.28, 0.54) | 0.5381 |
| ADHD | IL17RA | MR Egger | 26 | -0.2347 | 0.3575 | -0.23 (-0.93, 0.47) | 0.5178 |
| ADHD | IL17RA | Weighted median | 26 | 0.0368 | 0.1177 | 0.04 (-0.19, 0.27) | 0.7542 |
| ADHD | IL17RA | Weighted mode | 26 | 0.2187 | 0.2631 | 0.22 (-0.3, 0.74) | 0.4137 |
| ADHD | IL17F | MR Egger | 26 | -0.1654 | 0.3538 | -0.17 (-0.86, 0.52) | 0.6443 |
| ADHD | IL17F | Weighted median | 26 | -0.0819 | 0.1141 | -0.08 (-0.3, 0.14) | 0.4728 |
| ADHD | IL17F | Weighted mode | 26 | -0.1302 | 0.1945 | -0.13 (-0.51, 0.25) | 0.5094 |
| ADHD | IL22RA | MR Egger | 26 | 0.3639 | 0.343 | 0.36 (-0.31, 1.03) | 0.2994 |
| ADHD | IL22RA | Weighted median | 26 | 0.1091 | 0.1087 | 0.11 (-0.1, 0.32) | 0.3152 |
| ADHD | IL22RA | Weighted mode | 26 | 0.1053 | 0.2168 | 0.11 (-0.31, 0.53) | 0.6313 |
| ADHD | IL10RB | MR Egger | 26 | 0.4556 | 0.3447 | 0.46 (-0.22, 1.14) | 0.1988 |
| ADHD | IL10RB | Weighted median | 26 | -0.012 | 0.1145 | -0.01 (-0.23, 0.21) | 0.9165 |
| ADHD | IL10RB | Weighted mode | 26 | 0.0247 | 0.2294 | 0.02 (-0.43, 0.47) | 0.9153 |

Table S7B. Pleiotropy test derived from MR-Egger regression.

| exposure | outcome | Estimated intercept | Standard deviation | t statistic | P value |
| --- | --- | --- | --- | --- | --- |
| ADHD | IL22RA | -0.018904632 | 0.02254738 | -0.8384403 | 0.41005667 |
| ADHD | IFNGR1 | -0.025343938 | 0.01851492 | -1.3688386 | 0.18372629 |
| ADHD | IL10RB | -0.039317644 | 0.02330075 | -1.6873983 | 0.10448279 |
| ADHD | TGFB1 | -0.029183356 | 0.01997942 | -1.4606706 | 0.15707466 |
| ADHD | IL22RA | -0.01054409 | 0.022991 | -0.4586181 | 0.6506346 |
| ADHD | IL2 | -0.008168808 | 0.02373859 | -0.3441152 | 0.73375653 |
| ADHD | IL12RB2 | -0.01948083 | 0.02883277 | -0.675649 | 0.5057222 |
| ADHD | IL23R | -0.028431351 | 0.02089901 | -1.3604163 | 0.18633947 |
| ADHD | IL17RA | 0.014634905 | 0.02416537 | 0.60561467 | 0.55045549 |
| ADHD | IL21 | 0.034443169 | 0.02626847 | 1.31119797 | 0.202198 |
| ADHD | IL12RB1 | -0.026061152 | 0.02052775 | -1.2695574 | 0.21641527 |
| ADHD | IL5 | -0.026877255 | 0.03046711 | -0.8821729 | 0.38643382 |
| ADHD | IL4R | 0.013443131 | 0.02513262 | 0.53488776 | 0.59764886 |
| ADHD | IL6R | -0.018238432 | 0.02630345 | -0.6933854 | 0.49472413 |
| ADHD | IL13RA1 | 0.000135056 | 0.02581972 | 0.00523071 | 0.99586975 |
| ADHD | IL5RA | -0.041881705 | 0.0218965 | -1.9127126 | 0.06779038 |
| ADHD | IL17F | 0.00244129 | 0.02391013 | 0.10210274 | 0.91952342 |
| ADHD | IL9 | -0.0414834 | 0.01807656 | -2.2948729 | 0.03078697 |
| ADHD | IL12B | -0.013244122 | 0.02057531 | -0.6436902 | 0.52588019 |

Table S8. Genetic instruments used for the MR analysis investigating the causal effect of serum CRP on ADHD.

| CHR | BP | SNP | BETA | SE | P | A1 | A2 | F statistic |
| --- | --- | --- | --- | --- | --- | --- | --- | --- |
| 1 | 154418225 | rs531479718 | -0.09 | 0.00 | 4.94E-324 | CAA | C | 1534.60 |
| 12 | 121356075 | rs61946383 | 0.08 | 0.00 | 4.94E-324 | A | G | 1535.89 |
| 1 | 66070031 | rs11208685 | 0.10 | 0.00 | 2.25E-320 | A | T | 1517.88 |
| 2 | 27730940 | rs1260326 | 0.08 | 0.00 | 2.7E-303 | T | C | 1436.41 |
| 19 | 45337918 | rs147711004 | -0.21 | 0.01 | 9.15E-302 | A | G | 1379.41 |
| 1 | 159613986 | rs4656241 | -0.09 | 0.00 | 2.76E-264 | T | C | 1250.33 |
| 16 | 51436882 | rs17616063 | 0.13 | 0.00 | 3.91E-205 | A | G | 924.71 |
| 8 | 9173209 | rs7012637 | 0.05 | 0.00 | 1.29E-132 | A | G | 622.50 |
| 2 | 113841030 | rs6734238 | -0.04 | 0.00 | 9.53E-108 | A | G | 504.00 |
| 1 | 247601595 | rs12239046 | -0.04 | 0.00 | 1.34E-87 | T | C | 408.04 |
| 20 | 43042364 | rs1800961 | -0.11 | 0.01 | 6.495E-74 | T | C | 331.36 |
| 14 | 73011885 | rs2239222 | -0.04 | 0.00 | 2.853E-63 | A | G | 292.25 |
| 15 | 60883281 | rs339969 | 0.03 | 0.00 | 1.292E-56 | A | C | 260.59 |
| 21 | 40465066 | rs4817984 | -0.03 | 0.00 | 3.139E-53 | A | C | 244.50 |
| 22 | 39096602 | rs6519133 | 0.03 | 0.00 | 1.578E-48 | T | C | 222.15 |
| 1 | 27180088 | rs75460349 | 0.09 | 0.01 | 3.401E-45 | A | C | 200.20 |
| 8 | 116974302 | rs10095930 | 0.03 | 0.00 | 8.234E-45 | T | C | 204.49 |
| 11 | 13361524 | rs6486122 | 0.03 | 0.00 | 1.38E-43 | T | C | 198.68 |
| 15 | 53728710 | rs149624078 | -0.14 | 0.01 | 3.848E-43 | T | C | 188.93 |
| 1 | 91530432 | rs469882 | 0.03 | 0.00 | 8.684E-43 | A | C | 194.88 |
| 12 | 95857690 | rs12231235 | -0.03 | 0.00 | 9.943E-43 | A | G | 194.60 |
| 6 | 126851160 | rs1490384 | -0.03 | 0.00 | 9.943E-43 | T | C | 194.60 |
| 14 | 94844947 | rs28929474 | -0.10 | 0.01 | 1.674E-41 | T | C | 183.71 |
| 1 | 40035928 | rs3768321 | 0.03 | 0.00 | 3.057E-40 | T | G | 182.79 |
| 16 | 53806453 | rs56094641 | -0.03 | 0.00 | 5.351E-39 | A | G | 176.89 |
| 18 | 55089715 | rs55855238 | -0.03 | 0.00 | 1.478E-36 | T | C | 165.31 |
| 10 | 91011458 | rs1332328 | 0.03 | 0.00 | 2.807E-35 | T | C | 159.24 |
| 7 | 22759469 | rs1880241 | 0.03 | 0.00 | 3.544E-35 | A | G | 158.76 |
| 7 | 72895946 | rs75229905 | 0.04 | 0.00 | 4.016E-34 | T | C | 144.00 |
| 17 | 76357874 | rs11868378 | -0.03 | 0.00 | 5.735E-34 | A | G | 153.03 |
| 11 | 47245389 | rs4647725 | 0.03 | 0.00 | 8.943E-34 | T | C | 152.11 |
| 17 | 72702914 | rs1037170 | 0.03 | 0.00 | 3.563E-33 | T | C | 149.26 |
| 18 | 12783898 | rs2542153 | -0.02 | 0.00 | 2.613E-31 | T | C | 140.42 |
| 9 | 136149229 | rs505922 | -0.02 | 0.00 | 5.212E-29 | T | C | 129.53 |
| 7 | 36085142 | rs2700938 | -0.02 | 0.00 | 2.508E-28 | T | C | 126.29 |
| 15 | 43820717 | rs55707100 | 0.07 | 0.01 | 5.789E-28 | T | C | 120.65 |
| 20 | 44551855 | rs6073958 | 0.03 | 0.00 | 5.871E-28 | T | C | 124.55 |
| 19 | 49206145 | rs516316 | 0.02 | 0.00 | 6.544E-28 | C | G | 124.32 |
| 3 | 49941436 | rs2280406 | 0.02 | 0.00 | 1.124E-27 | A | G | 123.21 |
| 17 | 36073320 | rs17138478 | 0.03 | 0.00 | 9.234E-27 | A | C | 111.57 |
| 12 | 6440009 | rs1800693 | 0.02 | 0.00 | 9.556E-27 | T | C | 118.81 |
| 6 | 130373648 | rs7756870 | -0.02 | 0.00 | 1.106E-25 | C | G | 113.78 |
| 8 | 126500031 | rs28601761 | 0.02 | 0.00 | 3.689E-25 | C | G | 111.30 |
| 2 | 214033530 | rs1441169 | 0.02 | 0.00 | 6.157E-25 | A | G | 110.25 |
| 11 | 59936926 | rs7933202 | 0.02 | 0.00 | 6.157E-25 | A | C | 110.25 |
| 17 | 57911230 | rs1292061 | -0.02 | 0.00 | 1.025E-24 | A | G | 109.20 |
| 3 | 135932359 | rs687339 | -0.02 | 0.00 | 2.193E-24 | T | C | 107.64 |
| 2 | 169893419 | rs2161037 | 0.02 | 0.00 | 7.697E-24 | A | G | 105.06 |
| 9 | 139281847 | rs78428995 | -0.02 | 0.00 | 2.079E-23 | T | C | 103.02 |
| 12 | 103522952 | rs4764939 | -0.02 | 0.00 | 5.562E-23 | T | C | 101.00 |
| 2 | 632591 | rs13028310 | -0.03 | 0.00 | 9.064E-23 | T | C | 100.00 |
| 20 | 62370349 | rs1056441 | -0.02 | 0.00 | 2.283E-22 | T | C | 98.10 |
| 12 | 24195042 | rs11047224 | -0.05 | 0.01 | 3.903E-22 | C | G | 93.44 |
| 8 | 144643169 | rs1545536 | -0.02 | 0.00 | 9.143E-22 | T | C | 95.26 |
| 7 | 99181096 | rs11772470 | -0.03 | 0.00 | 1.006E-21 | A | G | 88.62 |
| 2 | 102757139 | rs4141632 | 0.02 | 0.00 | 1.683E-21 | A | G | 94.01 |
| 16 | 27325021 | rs12927172 | 0.02 | 0.00 | 2.589E-21 | A | G | 93.12 |
| 17 | 64228995 | rs149394327 | 0.06 | 0.01 | 5.768E-21 | C | G | 88.60 |
| 5 | 172176886 | rs2161374 | -0.02 | 0.00 | 6.604E-21 | T | C | 91.20 |
| 19 | 51728477 | rs12459419 | -0.02 | 0.00 | 8.426E-21 | T | C | 90.70 |
| 5 | 52080909 | rs77704739 | 0.05 | 0.01 | 1.38E-20 | T | C | 86.28 |
| 19 | 19379549 | rs58542926 | 0.04 | 0.00 | 3.528E-20 | T | C | 83.32 |
| 2 | 25130440 | rs11689543 | -0.02 | 0.00 | 6.58E-20 | A | T | 86.49 |
| 22 | 41409429 | rs9611454 | -0.02 | 0.00 | 7.491E-20 | T | C | 86.22 |
| 12 | 31997635 | rs9738365 | 0.02 | 0.00 | 1.388E-19 | A | C | 84.96 |
| 6 | 138006504 | rs6920220 | 0.02 | 0.00 | 3.175E-19 | A | G | 83.27 |
| 6 | 116327936 | rs4354188 | 0.02 | 0.00 | 9.599E-19 | T | C | 81.00 |
| 14 | 96933414 | rs55981844 | 0.02 | 0.00 | 9.599E-19 | T | C | 81.00 |
| 11 | 27700125 | rs7103411 | 0.02 | 0.00 | 9.599E-19 | T | C | 81.00 |
| 12 | 47198899 | rs2429473 | 0.02 | 0.00 | 1.363E-18 | A | C | 80.28 |
| 7 | 1028448 | rs3808348 | -0.02 | 0.00 | 1.363E-18 | T | C | 80.28 |
| 14 | 24874193 | rs72694393 | -0.02 | 0.00 | 1.488E-18 | T | G | 80.10 |
| 19 | 38229926 | rs11666245 | -0.04 | 0.00 | 2.021E-18 | A | G | 76.20 |
| 3 | 170695426 | rs1905505 | 0.02 | 0.00 | 2.126E-18 | A | G | 79.37 |
| 20 | 25262789 | rs2261790 | -0.02 | 0.00 | 2.301E-18 | T | C | 79.21 |
| 9 | 92207308 | rs7357754 | -0.02 | 0.00 | 3.55E-18 | A | G | 78.32 |
| 7 | 150294632 | rs35462231 | 0.02 | 0.00 | 4.1E-18 | A | T | 78.03 |
| 1 | 221103388 | rs11118625 | 0.02 | 0.00 | 4.672E-18 | A | G | 77.76 |
| 5 | 78570219 | rs6453434 | 0.02 | 0.00 | 7.576E-18 | T | G | 76.77 |
| 1 | 21820042 | rs12132412 | -0.02 | 0.00 | 1.705E-17 | A | G | 75.11 |
| 11 | 55371381 | rs75423534 | -0.03 | 0.00 | 2.013E-17 | A | G | 70.56 |
| 12 | 56929694 | rs2657896 | -0.02 | 0.00 | 2.332E-17 | T | C | 74.47 |
| 6 | 41671677 | rs4714508 | -0.02 | 0.00 | 5.666E-17 | A | G | 72.66 |
| 18 | 57851763 | rs10871777 | -0.02 | 0.00 | 5.764E-17 | A | G | 72.62 |
| 13 | 113927208 | rs9604045 | -0.02 | 0.00 | 8.057E-17 | T | G | 71.94 |
| 3 | 24520283 | rs6792725 | 0.02 | 0.00 | 1.465E-16 | A | G | 70.71 |
| 16 | 88528636 | rs60037105 | 0.02 | 0.00 | 3.084E-16 | A | T | 69.19 |
| 10 | 94839724 | rs4418728 | -0.02 | 0.00 | 3.575E-16 | T | G | 68.89 |
| 16 | 28831359 | rs4788095 | -0.02 | 0.00 | 3.575E-16 | T | C | 68.89 |
| 20 | 39142516 | rs2207132 | 0.05 | 0.01 | 5.18E-16 | A | G | 65.88 |
| 11 | 43628749 | rs4755720 | -0.02 | 0.00 | 5.36E-16 | T | C | 68.06 |
| 9 | 102281383 | rs10760691 | -0.02 | 0.00 | 8.654E-16 | A | G | 67.08 |
| 17 | 40476363 | rs8072566 | -0.02 | 0.00 | 1.105E-15 | A | G | 66.59 |
| 20 | 48960546 | rs6020459 | -0.02 | 0.00 | 1.914E-15 | T | C | 65.46 |
| 16 | 69565461 | rs12929503 | 0.02 | 0.00 | 2.645E-15 | T | C | 64.80 |
| 1 | 198442821 | rs6686560 | 0.03 | 0.00 | 3.919E-15 | A | G | 60.18 |
| 10 | 80819132 | rs704017 | 0.02 | 0.00 | 3.919E-15 | A | G | 64.00 |
| 11 | 72499035 | rs663015 | -0.02 | 0.00 | 5.686E-15 | T | C | 63.24 |
| 12 | 90462006 | rs7310135 | 0.02 | 0.00 | 5.792E-15 | T | C | 63.20 |
| 11 | 65931919 | rs7127808 | -0.02 | 0.00 | 7.142E-15 | A | T | 62.78 |
| 2 | 242370751 | rs59916403 | 0.02 | 0.00 | 8.232E-15 | T | G | 62.49 |
| 4 | 45121873 | rs7662792 | -0.02 | 0.00 | 8.232E-15 | A | T | 62.49 |
| 17 | 16083684 | rs178826 | 0.02 | 0.00 | 8.541E-15 | T | C | 62.41 |
| 16 | 83979317 | rs67890964 | -0.02 | 0.00 | 1.189E-14 | T | C | 61.73 |
| 19 | 41354533 | rs1801272 | 0.05 | 0.01 | 3.019E-14 | A | T | 58.11 |
| 16 | 2169458 | rs4018180 | -0.03 | 0.00 | 3.102E-14 | A | G | 56.97 |
| 19 | 35553341 | rs1688043 | -0.03 | 0.00 | 6.931E-14 | T | C | 55.34 |
| 1 | 214392881 | rs10864088 | -0.02 | 0.00 | 7.246E-14 | A | G | 58.05 |
| 10 | 30708441 | rs303429 | -0.02 | 0.00 | 8.354E-14 | T | C | 57.76 |
| 6 | 98421855 | rs543492421 | 0.02 | 0.00 | 1.082E-13 | A | AT | 57.23 |
| 20 | 57290705 | rs12220 | -0.02 | 0.00 | 1.212E-13 | T | C | 57.00 |
| 17 | 47135875 | rs68085814 | -0.02 | 0.00 | 2.447E-13 | A | G | 55.57 |
| 15 | 63791125 | rs62011286 | -0.02 | 0.00 | 2.959E-13 | A | G | 55.18 |
| 6 | 153365834 | rs1338071 | 0.02 | 0.00 | 3.41E-13 | C | G | 54.89 |
| 12 | 21710749 | rs10770829 | 0.02 | 0.00 | 3.643E-13 | T | C | 54.76 |
| 15 | 58683366 | rs1532085 | 0.01 | 0.00 | 5.234E-13 | A | G | 54.02 |
| 9 | 104111836 | rs11790458 | -0.02 | 0.00 | 5.903E-13 | A | G | 53.78 |
| 10 | 21830104 | rs11012732 | -0.02 | 0.00 | 8.31E-13 | A | G | 53.08 |
| 5 | 156738540 | rs2289852 | -0.03 | 0.00 | 8.715E-13 | A | G | 50.60 |
| 17 | 68257738 | rs9905884 | 0.02 | 0.00 | 1.352E-12 | A | C | 52.09 |
| 1 | 222098690 | rs12140498 | -0.02 | 0.00 | 1.442E-12 | T | C | 51.96 |
| 3 | 9480906 | rs2596937 | -0.02 | 0.00 | 1.442E-12 | T | C | 51.96 |
| 2 | 37059557 | rs13021775 | -0.01 | 0.00 | 1.53E-12 | C | G | 51.84 |
| 7 | 101864836 | rs385417 | 0.01 | 0.00 | 1.53E-12 | A | C | 51.84 |
| 17 | 1618363 | rs11078597 | 0.02 | 0.00 | 1.616E-12 | T | C | 51.73 |
| 8 | 103688381 | rs10106298 | 0.02 | 0.00 | 1.637E-12 | A | G | 51.70 |
| 5 | 72979436 | rs750344 | 0.02 | 0.00 | 1.74E-12 | A | G | 51.58 |
| 1 | 22681214 | rs34761529 | -0.02 | 0.00 | 2.029E-12 | T | C | 51.27 |
| 7 | 74073590 | rs73137144 | 0.02 | 0.00 | 2.119E-12 | A | G | 51.18 |
| 6 | 7112819 | rs6925389 | -0.01 | 0.00 | 3.195E-12 | A | G | 50.34 |
| 16 | 79745672 | rs562609617 | -0.02 | 0.00 | 3.554E-12 | G | GT | 50.13 |
| 1 | 243832560 | rs4658403 | -0.02 | 0.00 | 3.631E-12 | T | C | 50.08 |
| 7 | 28189411 | rs1635852 | 0.01 | 0.00 | 4.378E-12 | T | C | 49.70 |
| 2 | 178180703 | rs1869358 | 0.02 | 0.00 | 4.638E-12 | T | C | 49.59 |
| 1 | 236312326 | rs10924372 | -0.01 | 0.00 | 6.185E-12 | T | C | 49.00 |
| 21 | 33122575 | rs16988471 | -0.03 | 0.00 | 6.185E-12 | A | C | 47.02 |
| 12 | 69744014 | rs1800973 | 0.03 | 0.00 | 6.185E-12 | A | C | 46.75 |
| 4 | 3447156 | rs4690098 | 0.02 | 0.00 | 8.236E-12 | T | C | 48.42 |
| 11 | 30361670 | rs1222209 | -0.01 | 0.00 | 8.578E-12 | A | C | 48.34 |
| 11 | 61519986 | rs2956395 | -0.01 | 0.00 | 1.187E-11 | C | G | 47.68 |
| 5 | 150435645 | rs12516176 | -0.02 | 0.00 | 1.569E-11 | T | C | 47.11 |
| 12 | 12879254 | rs7956514 | -0.02 | 0.00 | 1.569E-11 | T | G | 47.11 |
| 1 | 109816863 | rs552693039 | -0.02 | 0.00 | 2.001E-11 | T | TC | 43.56 |
| 1 | 205216110 | rs17417252 | -0.02 | 0.00 | 2.131E-11 | A | G | 46.49 |
| 18 | 45663787 | rs12605964 | 0.01 | 0.00 | 4.262E-11 | T | C | 45.08 |
| 5 | 56194768 | rs166169 | -0.02 | 0.00 | 4.262E-11 | T | C | 42.03 |
| 2 | 174847693 | rs10497423 | -0.01 | 0.00 | 4.684E-11 | T | G | 44.89 |
| 2 | 88438050 | rs4246598 | 0.01 | 0.00 | 4.684E-11 | A | C | 44.89 |
| 3 | 4762734 | rs7639927 | -0.01 | 0.00 | 4.684E-11 | A | T | 44.89 |
| 13 | 42573980 | rs7993752 | -0.01 | 0.00 | 4.684E-11 | A | C | 44.89 |
| 1 | 177885762 | rs630372 | 0.02 | 0.00 | 4.82E-11 | A | G | 44.83 |
| 14 | 58759280 | rs34864350 | -0.03 | 0.00 | 5.04E-11 | A | G | 42.82 |
| 15 | 52002270 | rs72730950 | 0.03 | 0.00 | 6.181E-11 | A | G | 42.08 |
| 1 | 1564194 | rs3935032 | -0.02 | 0.00 | 6.417E-11 | T | C | 44.25 |
| 2 | 232324510 | rs12620844 | -0.01 | 0.00 | 6.509E-11 | T | C | 44.22 |
| 10 | 75554541 | rs7908825 | -0.01 | 0.00 | 6.509E-11 | C | G | 44.22 |
| 2 | 171940975 | rs930035 | -0.01 | 0.00 | 6.509E-11 | A | G | 44.22 |
| 5 | 170525393 | rs245767 | -0.01 | 0.00 | 9.575E-11 | A | G | 43.44 |
| 1 | 44060483 | rs583040 | -0.01 | 0.00 | 1.248E-10 | A | G | 42.90 |
| 4 | 38765720 | rs10008492 | 0.01 | 0.00 | 1.478E-10 | T | C | 42.56 |
| 4 | 18459828 | rs994596 | 0.01 | 0.00 | 1.478E-10 | T | C | 42.56 |
| 19 | 2671100 | rs59737437 | -0.02 | 0.00 | 1.498E-10 | T | C | 42.53 |
| 14 | 21691941 | rs11156891 | -0.02 | 0.00 | 1.515E-10 | A | T | 42.51 |
| 1 | 63117010 | rs112957492 | 0.01 | 0.00 | 2.007E-10 | T | C | 41.94 |
| 2 | 203136872 | rs570916461 | 0.02 | 0.00 | 2.249E-10 | A | AT | 41.71 |
| 11 | 16251251 | rs2030291 | 0.01 | 0.00 | 2.372E-10 | A | T | 41.60 |
| 10 | 99784552 | rs112213274 | -0.01 | 0.00 | 2.613E-10 | CCA | C | 41.41 |
| 3 | 94075026 | rs13066686 | -0.01 | 0.00 | 3.258E-10 | A | C | 40.96 |
| 3 | 176869498 | rs6443429 | 0.01 | 0.00 | 3.674E-10 | A | C | 40.72 |
| 18 | 60203855 | rs34284056 | -0.01 | 0.00 | 4.097E-10 | A | C | 40.50 |
| 2 | 230734531 | rs10498240 | 0.01 | 0.00 | 4.955E-10 | A | C | 40.11 |
| 10 | 133736636 | rs7084062 | -0.01 | 0.00 | 4.955E-10 | A | G | 40.11 |
| 18 | 23798857 | rs59427082 | -0.01 | 0.00 | 8.321E-10 | T | C | 39.06 |
| 7 | 6416615 | rs7455288 | 0.01 | 0.00 | 1.017E-09 | T | C | 38.66 |
| 19 | 11019581 | rs11667234 | 0.03 | 0.00 | 1.076E-09 | A | C | 36.99 |
| 2 | 59298298 | rs13416992 | 0.01 | 0.00 | 1.132E-09 | A | C | 38.44 |
| 1 | 68088439 | rs787488 | 0.01 | 0.00 | 1.266E-09 | A | G | 38.21 |
| 13 | 110368764 | rs12428361 | -0.01 | 0.00 | 1.328E-09 | T | C | 38.12 |
| 1 | 112315450 | rs197419 | -0.01 | 0.00 | 1.537E-09 | A | C | 37.82 |
| 5 | 132291157 | rs72799498 | 0.02 | 0.00 | 1.554E-09 | A | G | 37.80 |
| 21 | 37447711 | rs2156407 | 0.01 | 0.00 | 1.67E-09 | A | G | 37.65 |
| 8 | 73435156 | rs6993128 | -0.01 | 0.00 | 1.67E-09 | T | C | 37.65 |
| 7 | 44798315 | rs113585586 | 0.02 | 0.00 | 1.701E-09 | T | G | 35.23 |
| 5 | 153157229 | rs2926851 | 0.01 | 0.00 | 2.081E-09 | T | C | 37.21 |
| 6 | 35050506 | rs2177382 | -0.02 | 0.00 | 2.433E-09 | A | G | 36.89 |
| 2 | 165518799 | rs75265117 | -0.02 | 0.00 | 2.544E-09 | C | G | 34.47 |
| 3 | 33457493 | rs11928797 | -0.02 | 0.00 | 2.608E-09 | A | C | 34.56 |
| 12 | 100926084 | rs77738620 | -0.06 | 0.01 | 2.619E-09 | T | C | 35.28 |
| 4 | 103401723 | rs141936164 | 0.01 | 0.00 | 2.852E-09 | A | G | 36.57 |
| 3 | 156915895 | rs1969066 | 0.01 | 0.00 | 2.889E-09 | A | G | 36.55 |
| 7 | 105658927 | rs114947103 | -0.02 | 0.00 | 3.013E-09 | T | C | 36.46 |
| 1 | 98426476 | rs28582591 | 0.02 | 0.00 | 3.013E-09 | A | G | 36.46 |
| 4 | 148981496 | rs10027275 | -0.01 | 0.00 | 3.79E-09 | C | G | 36.00 |
| 12 | 11791029 | rs11054402 | -0.01 | 0.00 | 3.79E-09 | T | C | 36.00 |
| 14 | 75737110 | rs11624418 | 0.01 | 0.00 | 3.79E-09 | A | G | 36.00 |
| 19 | 807442 | rs123698 | -0.01 | 0.00 | 3.79E-09 | C | G | 36.00 |
| 5 | 158252438 | rs17056278 | -0.02 | 0.00 | 3.79E-09 | C | G | 34.18 |
| 1 | 150269545 | rs500812 | 0.01 | 0.00 | 3.79E-09 | T | G | 36.00 |
| 6 | 88367635 | rs2307377 | -0.02 | 0.00 | 4.449E-09 | A | G | 33.82 |
| 1 | 10125407 | rs7539725 | 0.02 | 0.00 | 4.649E-09 | A | G | 33.25 |
| 5 | 124139761 | rs6595549 | -0.02 | 0.00 | 4.76E-09 | C | G | 35.54 |
| 8 | 129131132 | rs200733118 | 0.03 | 0.00 | 4.877E-09 | A | C | 34.03 |
| 3 | 18758501 | rs6775319 | -0.01 | 0.00 | 4.961E-09 | A | T | 35.46 |
| 1 | 200334535 | rs946551 | -0.01 | 0.00 | 4.961E-09 | T | C | 35.46 |
| 4 | 89050026 | rs74904971 | -0.02 | 0.00 | 5.485E-09 | A | C | 33.15 |
| 11 | 32956492 | rs62618693 | -0.03 | 0.01 | 6.08E-09 | T | C | 33.69 |
| 11 | 11820449 | rs10831676 | 0.01 | 0.00 | 6.836E-09 | A | C | 34.81 |
| 14 | 35621393 | rs146424514 | 0.02 | 0.00 | 7.295E-09 | A | G | 34.68 |
| 3 | 71632266 | rs6801781 | 0.01 | 0.00 | 8.452E-09 | A | G | 34.38 |
| 8 | 22452357 | rs11782130 | 0.01 | 0.00 | 8.777E-09 | T | G | 34.31 |
| 17 | 27653016 | rs11653826 | 0.02 | 0.00 | 8.994E-09 | T | C | 32.33 |
| 17 | 62023791 | rs3785568 | 0.02 | 0.00 | 9.355E-09 | T | C | 34.18 |
| 13 | 86367973 | rs34633805 | 0.01 | 0.00 | 9.695E-09 | CT | C | 34.11 |
| 9 | 107559855 | rs4149349 | 0.01 | 0.00 | 9.695E-09 | CG | C | 34.11 |
| 9 | 132611051 | rs73672511 | -0.02 | 0.00 | 1.008E-08 | T | G | 31.87 |
| 9 | 71069835 | rs10868852 | 0.03 | 0.01 | 1.024E-08 | A | C | 32.87 |
| 11 | 119095761 | rs11217192 | 0.01 | 0.00 | 1.051E-08 | T | G | 33.94 |
| 6 | 18492350 | rs5017416 | 0.03 | 0.00 | 1.051E-08 | T | G | 32.51 |
| 4 | 55508495 | rs78716428 | -0.01 | 0.00 | 1.051E-08 | A | AC | 33.94 |
| 11 | 107243922 | rs113584870 | -0.02 | 0.00 | 1.08E-08 | A | G | 31.59 |
| 7 | 128573994 | rs7801838 | -0.01 | 0.00 | 1.1E-08 | T | C | 33.85 |
| 15 | 62329607 | rs189400382 | -0.01 | 0.00 | 1.156E-08 | A | G | 33.75 |
| 21 | 46494995 | rs9977825 | 0.01 | 0.00 | 1.156E-08 | T | C | 33.75 |
| 6 | 134260676 | rs459625 | 0.01 | 0.00 | 1.222E-08 | T | C | 33.64 |
| 12 | 46322449 | rs10880868 | 0.01 | 0.00 | 1.282E-08 | T | C | 33.54 |
| 11 | 69163441 | rs12270146 | 0.01 | 0.00 | 1.282E-08 | A | G | 33.54 |
| 17 | 3928975 | rs577034922 | -0.01 | 0.00 | 1.282E-08 | CA | C | 33.54 |
| 3 | 131971811 | rs34169656 | -0.01 | 0.00 | 1.35E-08 | CT | C | 33.44 |
| 11 | 10404252 | rs66553261 | -0.01 | 0.00 | 1.35E-08 | A | ACT | 33.44 |
| 1 | 78623626 | rs17391694 | 0.02 | 0.00 | 1.479E-08 | T | C | 31.14 |
| 2 | 228976534 | rs12613751 | -0.01 | 0.00 | 1.52E-08 | A | G | 33.20 |
| 3 | 35670150 | rs1470560 | 0.01 | 0.00 | 1.52E-08 | A | G | 33.20 |
| 15 | 78802869 | rs9788721 | -0.01 | 0.00 | 1.52E-08 | T | C | 33.20 |
| 16 | 29936654 | rs12716972 | -0.01 | 0.00 | 1.627E-08 | A | G | 33.06 |
| 20 | 60597623 | rs1886008 | 0.02 | 0.00 | 1.627E-08 | T | C | 31.09 |
| 2 | 72500685 | rs56045434 | -0.02 | 0.00 | 1.627E-08 | C | G | 30.82 |
| 3 | 119529113 | rs3732356 | -0.02 | 0.00 | 1.806E-08 | T | G | 31.31 |
| 2 | 66758912 | rs12621948 | 0.01 | 0.00 | 1.852E-08 | C | G | 32.80 |
| 9 | 127084074 | rs2274782 | 0.01 | 0.00 | 1.994E-08 | T | C | 32.65 |
| 9 | 14442595 | rs424539 | -0.01 | 0.00 | 1.994E-08 | C | G | 32.65 |
| 4 | 140900251 | rs72712556 | -0.01 | 0.00 | 1.994E-08 | A | G | 32.65 |
| 9 | 15852777 | rs13294945 | 0.01 | 0.00 | 2.063E-08 | T | G | 32.59 |
| 16 | 89848559 | rs6500446 | 0.01 | 0.00 | 2.163E-08 | A | G | 32.49 |
| 1 | 7739140 | rs6695390 | 0.01 | 0.00 | 2.163E-08 | T | G | 32.49 |
| 5 | 107348180 | rs288183 | 0.01 | 0.00 | 2.259E-08 | T | G | 32.40 |
| 6 | 136023746 | rs12191817 | -0.01 | 0.00 | 2.611E-08 | T | G | 32.11 |
| 6 | 149973764 | rs12523793 | -0.01 | 0.00 | 2.611E-08 | A | G | 32.11 |
| 3 | 25363163 | rs11711864 | 0.01 | 0.00 | 2.867E-08 | A | G | 31.92 |
| 7 | 115907289 | rs17138528 | 0.01 | 0.00 | 3.095E-08 | T | C | 31.77 |
| 17 | 65903629 | rs4468667 | -0.01 | 0.00 | 3.095E-08 | A | G | 31.77 |
| 17 | 38086736 | rs145604770 | -0.03 | 0.01 | 3.254E-08 | A | G | 30.46 |
| 17 | 7079846 | rs142122735 | 0.01 | 0.00 | 3.299E-08 | CCACA | C | 31.64 |
| 6 | 111904528 | rs174373 | 0.01 | 0.00 | 3.299E-08 | T | C | 31.64 |
| 19 | 36276086 | rs2239949 | -0.01 | 0.00 | 3.41E-08 | T | C | 31.57 |
| 18 | 40776606 | rs9965184 | 0.01 | 0.00 | 3.41E-08 | A | T | 31.57 |
| 3 | 123065778 | rs11708067 | -0.01 | 0.00 | 3.613E-08 | A | G | 31.46 |
| 12 | 63455347 | rs17098829 | -0.01 | 0.00 | 3.613E-08 | C | G | 31.46 |
| 3 | 151997386 | rs35415272 | 0.01 | 0.00 | 3.613E-08 | A | AAAC | 31.46 |
| 9 | 29717279 | rs10969334 | -0.01 | 0.00 | 3.793E-08 | A | C | 31.36 |
| 2 | 121306440 | rs17050272 | 0.01 | 0.00 | 3.793E-08 | A | G | 31.36 |
| 3 | 36998755 | rs6768108 | -0.01 | 0.00 | 3.793E-08 | T | C | 31.36 |
| 6 | 166329862 | rs991946 | 0.01 | 0.00 | 3.793E-08 | T | C | 31.36 |
| 5 | 92611208 | rs56821385 | -0.04 | 0.01 | 3.856E-08 | T | C | 30.41 |
| 13 | 103410518 | rs76620584 | 0.02 | 0.00 | 4.049E-08 | A | G | 29.47 |
| 5 | 112915702 | rs10078214 | 0.01 | 0.00 | 4.601E-08 | A | C | 30.97 |
| 16 | 1129010 | rs4988483 | 0.03 | 0.01 | 4.736E-08 | A | C | 29.71 |

Table S9. Genetic instruments used for MR analysis investigating the causal effect of genetically proxied plasma cytokines on ADHD.

| EXPOSURE | EXPOSURE NAME | SNP | CHR | BP | A1 | A2 | EAF | B | SE | P | CIS/TRANS | F | STUDY |
| --- | --- | --- | --- | --- | --- | --- | --- | --- | --- | --- | --- | --- | --- |
| IL2 | Interleukin-2 | rs4241819 | 4 | 186235986 | T | C | 0.50721 | 0.1919 | 0.0247 | 7.10E-15 | trans | 60 | Sun et al. |
| IL12B | Interleukin-23 | rs4921484 | 5 | 158769753 | C | T | 0.678009 | 0.3123 | 0.0262 | 7.20E-33 | cis | 142 | Sun et al. |
| IL12B | Interleukin-23 | rs9815073 | 3 | 188115682 | A | C | 0.374252 | 0.2146 | 0.0277 | 8.70E-15 | trans | 60 | Sun et al. |
| IL12RB1 | Interleukin-12 Receptor Subunit Beta-1 | rs376008 | 19 | 18189568 | T | C | 0.33501 | -0.7569 | 0.039813 | 6.28E-69 | cis | 361 | Suhre et al. |
| IL12RB2 | Interleukin-12 Receptor Subunit Beta-2 | rs12566098 | 1 | 67889571 | G | C | 0.688055 | 0.2568 | 0.0267 | 6.00E-22 | cis | 93 | Sun et al. |
| IFNGR1 | Interferon Gamma Receptor 1 | rs7080536 | 10 | 115348046 | A | G | 0.043842 | 0.6262 | 0.0617 | 3.50E-24 | trans | 103 | Sun et al. |
| IL4RA | Interleukin-4 receptor subunit alpha | rs10418046 | 19 | 54327869 | G | T | 0.21727 | -0.1694 | 0.0298 | 1.38E-08 | trans | 32 | Sun et al. |
| IL5 | Interleukin-5 | rs704 | 17 | 26694861 | A | G | 0.466647 | -0.2887 | 0.0242 | 6.90E-33 | trans | 142 | Sun et al. |
| IL5RA | Interleukin-5 Receptor Subunit Alpha | rs77400868 | 3 | 3150964 | G | A | 0.13908 | 0.5096 | 0.0362 | 6.80E-45 | cis | 198 | Sun et al. |
| IL13RA1 | Interleukin-13 Receptor Subunit Alpha-1 | rs4241818 | 4 | 187153786 | C | T | 0.513587 | 0.1924 | 0.0246 | 5.10E-15 | trans | 61 | Sun et al. |
| TGFB1 | Transforming Growth Factor Beta 1 | rs1800470 | 19 | 41858921 | A | G | 0.621 | 0.259 | 0.024 | 4.50E-26 | cis | 116 | Emilsson et al. |
| IL9 | Interleukin-9 | No instruments (p<=5e-08) available in datasets | | | | | | | | | | | |
| IL6R | Interleukin‐6 Receptor Subunit Alpha | rs4129267 | 1 | 154426264 | T | C | 0.36 | 0.81 | 0.023272 | 2E‐265 | cis | 1211 | Folkersen et al. |
| IL21 | Interleukin-21 | rs12368181 | 12 | 7181105 | G | A | 0.13305 | -0.3688 | 0.0362 | 2.00E-24 | trans | 104 | Sun et al. |
| IL23R | Interleukin-23 Receptor | rs11581607 | 1 | 67707690 | A | G | 0.066948 | -0.42 | 0.0491 | 1.20E-17 | cis | 73 | Sun et al. |
| IL17RA | Interleukin-17 Receptor A | rs4819959 | 22 | 17586631 | A | G | 0.49643 | 0.9127 | 0.0195 | 1.00E-200 | cis | 2190 | Sun et al. |
| IL17F | Interleukin-17 F | rs9274952 | 6 | 32641868 | G | T | 0.36485 | 0.1677 | 0.03 | 2.40E-08 | trans | 31 | Sun et al. |
| IL22RA1 | Interleukin-22 Receptor Subunit Alpha-1 | rs1065853 | 19 | 45413233 | T | G | 0.077779 | -0.3498 | 0.0461 | 3.20E-14 | trans | 58 | Sun et al. |
| IL10RB | Interleukin 10 Receptor Subunit Beta | rs2834167 | 21 | 34640788 | A | G | 0.732 | 0.16 | 0.028 | 1.10E-08 | cis | 33 | Emilsson et al. |

Table S10. Genetic instruments used in the MR analyses investigating brain-specific effects of genetically predicted expression of genes encoding cytokines on ADHD.

| Exposure NAME | Exposure | SNP | CHR | BP | A1 | A2 | B | SE | P | EAF | F |
| --- | --- | --- | --- | --- | --- | --- | --- | --- | --- | --- | --- |
| Interleukin-2 receptor subunit alpha | IL2RA | rs12722497 | 10 | 6053965 | C | A | -0.36517 | 0.043232 | 3.00E-17 | 8.89E-01 | 71 |
| Interleukin-12A | IL12A | rs1353248 | 3 | 159905770 | C | T | 0.24656 | 0.029559 | 7.34E-17 | 6.97E-01 | 70 |
| Interleukin-12B | IL12B | rs75259819 | 5 | 158974924 | A | G | -0.21666 | 0.049462 | 1.18E-05 | 9.16E-01 | 19 |
| Interleukin-12 receptor subunit beta 1 | IL12RB1 | rs2644777 | 19 | 18067806 | A | C | 0.25866 | 0.027514 | 5.40E-21 | 6.84E-01 | 88 |
| Interleukin-12 receptor subunit beta 2 | IL12RB2 | rs72678518 | 1 | 67305714 | A | G | -0.27044 | 0.034361 | 3.53E-15 | 7.93E-01 | 62 |
| Interferon gamma receptor 1 | IFNGR1 | rs4896249 | 6 | 137272932 | C | T | -0.32471 | 0.044829 | 4.38E-13 | 8.97E-01 | 52 |
| Interferon gamma receptor 2 | IFNGR2 | rs9976971 | 21 | 33395791 | A | G | -0.1225 | 0.026135 | 2.77E-06 | 4.30E-01 | 22 |
| Interleukin-4 | IL4 | rs6879672 | 5 | 132690255 | A | G | -0.32098 | 0.029369 | 8.36E-28 | 2.51E-01 | 119 |
| Interleukin-4 receptor | IL4R | rs7205510 | 16 | 27310077 | G | A | 0.191158 | 0.030116 | 2.19E-10 | 2.90E-01 | 40 |
| Interleukin-5 | IL5 | rs2070730 | 5 | 132484108 | G | A | -0.28132 | 0.027541 | 1.71E-24 | 6.88E-01 | 104 |
| Interleukin-5 receptor subunit alpha | IL5RA | rs6768065 | 3 | 3068553 | T | A | -0.13574 | 0.028282 | 1.59E-06 | 4.55E-01 | 23 |
| Interleukin-13 | IL13 | rs12652920 | 5 | 132549548 | G | C | 0.180025 | 0.03224 | 2.35E-08 | 7.93E-01 | 31 |
| Interleukin-9 | IL9 | rs4487482 | 5 | 135866082 | A | G | 0.232605 | 0.034617 | 1.82E-11 | 8.08E-01 | 45 |
| Tranforming Growth Factor Beta 1 | TGFB1 | rs75520557 | 19 | 40491081 | A | G | 0.267952 | 0.060981 | 1.11E-05 | 9.47E-01 | 19 |
| Interleukin-6 | IL6 | rs2905346 | 7 | 22578629 | G | A | -0.1225 | 0.027445 | 8.06E-06 | 4.92E-01 | 20 |
| Interleukin-21 receptor | IL21 | rs35913539 | 16 | 27467908 | T | C | 0.612885 | 0.033774 | 1.36E-73 | 8.42E-01 | 329 |
| Interleukin-17 receptor A | IL17RA | rs2845391 | 22 | 17045798 | A | T | -0.50932 | 0.026428 | 9.23E-83 | 3.90E-01 | 371 |
| Interleukin-23 subunit A | IL23A | rs59917308 | 12 | 56264924 | C | T | -0.43832 | 0.051091 | 9.55E-18 | 9.32E-01 | 74 |
| Interleukin-10 receptor subunit beta | IL10RB | rs2834167 | 21 | 33268483 | A | G | -0.74472 | 0.027437 | ######## | 0.730871 | 737 |

A1: effect allele; A2: reference allele

Table S11. Proxied SNPs (instruments) identified by LD.

| phenotype | original SNP | proxied SNP | CHR | BP | Effect allele | Other allele | Beta | SE | P value |
| --- | --- | --- | --- | --- | --- | --- | --- | --- | --- |
| serum CRP | rs147711004 | rs41289512 | 19 | 45351516 | C | G | 0.1916 | 0.0052 | 4.72E-298 |
|  | rs505922 | rs687289 | 9 | 136137106 | A | G | 0.0232 | 0.0021 | 1.976E-27 |
|  | rs13028310 | rs13022337 | 2 | 632609 | A | G | -0.0256 | 0.0026 | 4.014E-22 |
|  | rs11689543 | rs6730191 | 2 | 25130920 | A | G | -0.0181 | 0.002 | 6.177E-19 |
|  | rs72694393 | rs72694391 | 14 | 24874026 | T | C | 0.0178 | 0.002 | 2.301E-18 |
|  | rs35462231 | rs13223512 | 7 | 150294683 | A | G | -0.0207 | 0.0024 | 2.425E-17 |
|  | rs11118625 | rs11118626 | 1 | 221108912 | C | G | -0.0189 | 0.0022 | 3.231E-17 |
|  | rs10770829 | rs1871141 | 12 | 21713150 | A | C | -0.0173 | 0.0025 | 1.07E-11 |
|  | rs12605964 | rs4076364 | 18 | 45665107 | A | C | -0.0137 | 0.0021 | 1.478E-10 |
|  | rs112957492 | rs12047226 | 1 | 63105538 | T | C | 0.0133 | 0.0021 | 4.955E-10 |
|  | rs10027275 | rs11099675 | 4 | 148985104 | T | C | 0.0126 | 0.0023 | 7.421E-08 |
|  | rs946551 | rs2050770 | 1 | 200333925 | A | G | 0.0123 | 0.0022 | 3.989E-08 |
|  | rs146424514 | rs77477310 | 14 | 35615793 | A | G | -0.0158 | 0.0027 | 9.051E-09 |
|  | rs34633805 | rs1337267 | 13 | 86364396 | T | C | -0.0114 | 0.0022 | 3.59E-07 |
|  | rs4149349 | rs2740479 | 9 | 107563437 | A | G | 0.011 | 0.0021 | 2.678E-07 |
|  | rs10868852 | rs11140670 | 9 | 70998967 | C | G | -0.0282 | 0.007 | 0.00005966 |
|  | rs11217192 | rs721444 | 11 | 119144243 | A | C | -0.0121 | 0.0022 | 6.589E-08 |
|  | rs78716428 | rs7684939 | 4 | 55509189 | A | G | -0.0107 | 0.002 | 1.483E-07 |
|  | rs189400382 | rs35967150 | 15 | 62324471 | A | G | 0.0109 | 0.002 | 8.655E-08 |
|  | rs34169656 | rs1395004 | 3 | 131966575 | A | C | -0.0111 | 0.002 | 5.005E-08 |
|  | rs66553261 | rs11042769 | 11 | 10405452 | A | G | -0.0104 | 0.002 | 3.267E-07 |
|  | rs13294945 | rs11515071 | 9 | 15855545 | T | C | -0.0094 | 0.0021 | 0.000011 |
|  | rs35415272 | rs1426385 | 3 | 151998053 | A | G | 0.01 | 0.002 | 9.064E-07 |
| plasma cytokine | rs10418046 | rs34436714 | 19 | 54327313 | A | C | -0.1696 | 0.0301 | 1.6982E-08 |
|  | rs1065853 | rs7412 | 19 | 45412079 | T | C | -0.3494 | 0.0461 | 3.47E-14 |

Table S12. Sensitivity analysis for causal effect of serum CRP on ADHD.

Supplementary Table 12A. Pleiotropy test (MR Egger regression).

| exposure | outcome | estimated intercept | SE for intercept | t statistic | P for intercept |
| --- | --- | --- | --- | --- | --- |
| serum CRP | ADHD | 0.005 | 0.002 | 2.95 | 0.0035 |

Supplementary Table 12B. Heterogeneity test after Steiger filtering.

| exposure | outcome | method | No. SNP | OR (95% CI) | P value |
| --- | --- | --- | --- | --- | --- |
| serum CRP | ADHD | MR Egger | 234 | 0.97 (0.87-1.07) | 0.5464 |
| serum CRP | ADHD | Weighted median | 234 | 1.03 (0.95-1.12) | 0.4714 |
| serum CRP | ADHD | Inverse variance weighted | 234 | 1.08 (1.01-1.16) | 0.0308 |
| serum CRP | ADHD | Weighted mode | 234 | 1.01 (0.94, 1.08) | 0.868 |

Supplementary Table 12C. Pleiotropy test after Steiger filtering (MR Egger regression).

| exposure | outcome | estimated intercept | SE for intercept | t statistic | P for intercept |
| --- | --- | --- | --- | --- | --- |
| serum CRP | ADHD | 0.004 | 0.002 | 2.509 | 0.0128 |
